# Improved micronutrient status and health outcomes in low- and middle-income countries following large-scale fortification: evidence from a systematic review and meta-analysis

**DOI:** 10.1093/ajcn/nqz023

**Published:** 2019-04-17

**Authors:** Emily C Keats, Lynnette M Neufeld, Greg S Garrett, Mduduzi N N Mbuya, Zulfiqar A Bhutta

**Affiliations:** 1 Centre for Global Child Health, Hospital for Sick Children, Toronto, Canada; 2 Global Alliance for Improved Nutrition, Geneva, Switzerland; 3 Center of Excellence in Women and Child Health, Aga Khan University, Karachi, Pakistan; 4 Dalla Lana School of Public Health, University of Toronto, Toronto, Canada

**Keywords:** fortification, developing countries, iron, vitamin A, folic acid, iodine, effectiveness, micronutrient status, functional outcomes, systematic review

## Abstract

**Background:**

Micronutrient malnutrition is highly prevalent in low- and middle-income countries (LMICs) and disproportionately affects women and children. Although the effectiveness of large-scale food fortification (LSFF) of staple foods to prevent micronutrient deficiencies in high-income settings has been demonstrated, its effectiveness in LMICs is less well characterized. This is important as food consumption patterns, potential food vehicles, and therefore potential for impact may vary substantially in these contexts.

**Objectives:**

The aim of this study was to determine the real-world impact of LSFF with key micronutrients (vitamin A, iodine, iron, folic acid) on improving micronutrient status and functional health outcomes in LMICs.

**Methods:**

All applicable published/unpublished evidence was systematically retrieved and analyzed. Studies were not restricted by age or sex. Meta-analyses were performed for quantitative outcomes and results were presented as summary RRs, ORs, or standardized mean differences (SMDs) with 95% CIs.

**Results:**

LSFF increased serum micronutrient concentrations in several populations and demonstrated a positive impact on functional outcomes, including a 34% reduction in anemia (RR: 0.66; 95% CI: 0.59, 0.74), a 74% reduction in the odds of goiter (OR: 0.26; 95% CI: 0.16, 0.43), and a 41% reduction in the odds of neural tube defects (OR: 0.59; 95% CI: 0.49, 0.70). Additionally, we found that LSFF with vitamin A could protect nearly 3 million children per year from vitamin A deficiency. We noted an age-specific effect of fortification, with women (aged >18 y) attaining greater benefit than children, who may consume smaller quantities of fortified staple foods. Several programmatic/implementation factors were also reviewed that may facilitate or limit program potential.

**Conclusions:**

Measurable improvements in the micronutrient and health status of women and children are possible with LSFF. However, context and implementation factors are important when assessing programmatic sustainability and impact, and data on these are quite limited in LMIC studies.

## Background

Micronutrient malnutrition, caused by a deficiency in ≥1 essential vitamins and minerals, is widely prevalent and significantly associated with the global burden of poverty and disease. Although global control of iodine deficiency appears to be within reach (1), low iron, vitamin A, and zinc remain to be significant risk factors for poor health outcomes (2–4). Pregnant and lactating women and children are the most vulnerable (5, 6), particularly those living in low- and middle-income countries (LMICs) where diets consist largely of staple foods that do not provide enough nutritional value to reach the recommended daily intake of these essential micronutrients.

Food fortification is a strategy that has been used safely and effectively to prevent micronutrient deficiencies in high-income countries for more than a century. Large-scale food fortification (LSFF) is defined as the mandatory or voluntary addition of essential micronutrients to widely consumed staple foods and condiments during production (7, 8). It is an especially suitable solution where there is evidence of micronutrient deficiency at a population level. It is becoming an increasingly attractive investment in LMICs for several additional reasons, including rapid urbanization and increasing household purchasing power, leading to a greater proportion of the population relying on centrally processed foods (9, 10).

To generate the best evidence to inform global nutrition guidelines, it is imperative that current research (both research implementation and the interpretation of research through the use of systematic reviews) adapts to answer questions that will be most relevant to people and health systems. The past distinction between efficacy and effectiveness studies is becoming blurred (11), and there is merit in looking beyond randomized controlled trials (RCTs), where intervention acceptance and availability are maximal. For a complex intervention such as LSFF that relies heavily on functional programmatic elements, closing the efficacy-effectiveness or research-practice gap is necessary to determine how the intervention will fare under less-optimized conditions (11). In conducting LSFF research, process evaluations should be used to provide mechanistic evidence by measuring indicators along the impact pathway. In related systematic reviews, this refers to more inclusive evidence selection processes (i.e., from large-scale programs, especially given the need to reach people at scale) and the incorporation of biological and social mechanistic theory (11) when conducting subgroup analyses or interpreting findings.

Existing reviews on fortification strategies for children in LMICs (12–15) have demonstrated improved micronutrient status and reductions in anemia prevalence, but lack consensus regarding the benefits of fortification for improving functional health outcomes and mortality. These reviews are generally limited to specific subsets of populations and single-food vehicles, and are typically based on controlled trials only. The current evidence has yet to be analyzed to incorporate findings from programmatic settings or large-scale evaluations conducted in LMIC community settings and that skew more towards the effectiveness rather than the efficacy side of the spectrum (11).

Consequently, the main objective of this review was to determine the real-world impact of LSFF of staple foods with key micronutrients (iron, folic acid, vitamin A, and iodine) on health and nutrition outcomes in LMICs.

## Methods

### Protocol and registration

The protocol for this review has been accepted by the Campbell Collaboration (IDNG1401).

### Data retrieval

All applicable published and unpublished evidence on the effectiveness of LSFF in LMICs was systematically retrieved and analyzed. See the online Supplemental Material for a complete list of data sources (**Supplemental Table 1**) and details of the search strategy (**Supplemental Table 2**). Study inclusion/exclusion criteria are summarized in **Table 1**. There was no restriction of the study population by age or sex. Key micronutrients were selected for evaluation based on the literature surrounding prevalent micronutrient deficiencies and established food fortification programs in LMICs, along with evidence to suggest intervention efficacy (12–15). Programmatic knowledge pertaining to sector priorities and program relevance of the selection were provided by experts in the field (from the Global Alliance for Improved Nutrition). The inclusion of food as a “staple” was established in a context-specific manner, based on consumption habits of a population (e.g., soy sauce is considered a staple food across Asia). In order to capture effectiveness (i.e., impact in a real-world setting), only studies evaluating existing LSFF programs or voluntary efforts that had been taken to scale were considered for inclusion. As such, any small-scale (<1000 participants per arm) randomized or quasi-randomized trial was excluded. The date of the final search was November 5, 2018. There were no restrictions regarding date of publication. All screening (title/abstract and full text) and quality assessment was conducted in duplicate. An independent reviewer resolved any disputes. The data abstraction was also performed in duplicate, following piloting and standardization of the data abstraction form. Authors were contacted in the event of missing information or where data required clarification.

**TABLE 1:** Study inclusion and exclusion criteria^1^

Inclusion criteria	Exclusion criteria
Study population classified as LMIC (defined by World Bank cut-offs at the time of search)	Bioavailability studies
Food vehicle is considered a staple food or condiments	Small-scale efficacy trials (<1000 individuals per arm)
Fortification with 1 of the following micronutrients, alone or in combination: iron, folic acid, iodine, vitamin A	Studies pertaining to other fortification interventions, including targeted fortification (i.e., fortified foods designed for certain population subsets), home fortification with micronutrient powders, and biofortification
Observational (cohort, longitudinal, case-controlled, cross-sectional, before/after, qualitative) studies or gray literature documents (e.g., government documents) that evaluate an existing national/subnational fortification program	Studies looking at non-staple foods, including blended foods, complementary foods, and highly-processed foods
Experimental (randomized or quasi-randomized controlled trials) studies that are described as “large-scale” (i.e., implementation unit is >1000 participants)	—
English language	—

^1^LMICs, low- or middle-income countries.

### Data synthesis and analysis

We set out to evaluate a range of quantitative outcomes, including serum micronutrient levels, hematologic markers, and functional/clinical health outcomes. Primary outcomes of interest included serum retinol, anemia, goiter, and neural tube defects (NTDs). Secondary outcomes included serum/plasma folate, red blood cell folate, serum ferritin, hemoglobin concentration, urinary iodine concentration, acute malnutrition (weight-for-height), stunting (height-for-age), underweight (weight-for-age), hypothyroidism, hyperthyroidism, night blindness, xerophthalmia, adverse pregnancy outcomes, neurologic impairment, cognitive dysfunction, morbidity, and mortality. We found that several outcomes were not reported in the literature and, based on data availability, only some could be pooled (**Supplemental Table 3**). Definitions of included outcomes are listed in **Table 2**. Where data allowed (≥2 studies reporting an outcome), meta-analyses were performed for quantitative outcomes and results were presented as RRs, ORs, or standard mean differences (SMDs) and 95% CIs. For studies with multiple repeated measurements, the most recent data was used from both prefortification and postfortification periods. For example, if a mandatory fortification program was implemented in 1993 and measures were taken in 1990, 1992, 1994, and 1996, data from 1992 and 1996 would be included within the meta-analysis. Where applicable, estimates were combined to produce a weighted average that could be included in the meta-analysis (e.g., if a study provided regional estimates only, or if data on children had been disaggregated by sex). Intervention duration was calculated based on the year of LSFF implementation and the final year of data collection for each study. If there was significant revitalization of a fortification program (e.g., partnership with a nongovernmental organization that provided financial, technical, operational, logistic, and commodity support), then we have used this more recent date as the year of implementation. Subgroup analyses were performed according to age group for all outcomes. The range of each age group differed depending on the data available per outcome (i.e., was not prespecified), although we attempted to capture preschool-age children (1–4 y), school-age children (5–9 y), adolescents (10–19 y), and women of reproductive age (WRA), where possible. Random effects models were used to account for between-study heterogeneity. Heterogeneity was examined based on the *I*^2^ statistic (*I*^2^ > 50 indicating moderate heterogeneity) and a chi-squared *P* value <0.1, and through visual inspection of forest plots. Review Manager Software version 5.3 was utilized.

**TABLE 2:** Definitions of outcomes examined

Term	Definition
Vitamin A deficiency	Children: serum retinol level <0.7 μmol/L
	Lactating mothers: serum retinol level <1.05 μmol/L
Iodine deficiency	Mild: median urinary iodine for school-aged children 50–99 μg/L
	Moderate: median urinary iodine 20–49 μg/L
	Severe: median urinary iodine <20 μg/L
	Adequate iodine nutrition: median urinary iodine 100–199 μg/L
	Risk of adverse health consequences: median urinary iodine ≥300 μg/L
Goiter	Grade 0: no palpable or visible goiter
	Grade 1: a goiter that is palpable but not visible when the neck is in the normal position
	Grade 2: swelling in the neck that is clearly visible when the neck is in the normal position; consistent with an enlarged thyroid gland
Anemia	Children 6–59 mo: hemoglobin <110 g/L
	Children 5–11 y: hemoglobin <115 g/L
	Children 12–14 y: hemoglobin <120 g/L
	Women ≥15 y: hemoglobin <120 g/L
	Pregnant women: hemoglobin <110 g/L
Iron deficiency	Children <5 y: depleted iron stores <12 μg/L
	Children ≥5 y: depleted iron stores < 15 μg/L
	Severe risk of iron overload (adult males): >200 μg/L
	Severe risk of iron overload (adult females): >150 μg/L
Folate deficiency	Serum folate level: <10 nmol/L

### Quality assessment of evidence

Quality assessment of individual studies and the body of evidence for each outcome was determined through the use of the Child Health Epidemiology Research Group (CHERG) grading system, an extension of the guidelines developed by the Cochrane Collaboration and the Working Group for Grading of Recommendations Assessment, Development and Evaluation (GRADE). The CHERG approach uses 4 categories of criteria for assessing quality of evidence of individual studies: *1*) study design; *2*) study quality; *3*) relevance to the objectives of the review; *4*) consistency across studies (16). An overall grade (high, moderate, low, or very low) was then assigned for each outcome, based on the following criteria: *1*) volume and consistency of evidence; *2*) effect size; and *3*) strength of statistical evidence, as reflected by the *P* value. CHERG scores are reported alongside results (**Tables 3** –**6**), and full quality assessment tables can be found in the online Supplemental Material (**Supplemental Tables 4–7**).

**TABLE 3:** Summary of results for LSFF with vitamin A^1^

		Age groups			
Outcome	Combined effect	Children <12 mo	Children 12–59 mo	Children 5–9 y	WRA
Serum retinol, μg/dL	SMD: 0.31 (95% CI: 0.18, 0.45)	SMD: 0.31 (95% CI: 0.16, 0.46)	SMD: 0.14 (95% CI: 0.02, 0.25)	SMD: 0.50 (95% CI: 0.16, 0.85)	SMD: 0.45 (95% CI: 0.26, 0.64)
Study population	4 studies, *n* = 2800	1 study, *n* = 699	3 studies, *n* = 1163	2 studies, *n* = 491	1 study, *n* = 447
LSFF duration	12–24 mo	12 mo	12–24 mo	12 mo	12 mo
CHERG score	Moderate	NA	Moderate	Low	NA

^1^Analysis model: random effects; statistical method: inverse-variance. CHERG, Child Health Epidemiology Research Group; LSFF, large-scale food fortification; NA, not applicable; SMD, standard mean difference; WRA, women of reproductive age.

**TABLE 4:** Summary of results for salt iodization^1^

		Age groups	
Outcome	Combined effect	School-age children	WRA
Urinary iodine, μg/L	SMD: 1.02 (95%: CI: 0.63, 1.42)	SMD: 1.12 (95% CI: 0.57, 1.67)	SMD: 0.65 (95% CI: 0.54, 0.75)
Study population	4 studies, *n* = 8863	4 studies, *n* = 6871	1 study, *n* = 1992
LSFF duration	10 mo–6 y	10 mo–6 y	6 y
CHERG score	Low	Low	N/A
Iodine deficiency	RR: 0.25 (95% CI: 0.21, 0.29)	RR: 0.25 (95% CI: 0.21, 0.29)	RR: 0.26 (95% CI: 0.15, 0.45)
Study population	3 studies, *n* = 2423	3 studies, *n* = 2301	1 study, *n* = 122
LSFF duration	12 mo–3 y	12 mo–3 y	3 y
CHERG score	Low	Low	NA
Goiter prevalence	OR: 0.26 (95% CI: 0.16, 0.43)	OR: 0.26 (95% CI: 0.16, 0.43)	—
Study population	8 studies, *n* = 25,762	8 studies, *n* = 25,762	—
LSFF duration	12 mo–14 years	12 mo–14 y	—
CHERG score	Moderate	Moderate	—

^1^Analysis model: random effects; Statistical method: Mantel-Haenszel (dichotomous outcomes) and inverse-variance (continuous outcomes). CHERG, Child Health Epidemiology Research Group; LSFF, large-scale food fortification; NA, not applicable; SMD, standard mean difference; WRA, women of reproductive age.

**TABLE 5:** Summary of results for LSFF with iron, by age group and status^1^

		Age groups						
Outcome	Combined effect^2^	Children <7 y	Children 6–18 y	WRA	Pregnant women	Anemic children 1–5 y	Anemic children 5–10 y	Anemic WRA
Hb concentration, g/dL	SMD : 0.19 (95% CI: 0.04, 0.35)	SMD : 0.30 (95% CI: –0.05, 0.66)	SMD : 0.13 (95% CI: –0.08, 0.35)	SMD : 0.15 (95% CI: –0.08, 0.37)	SMD : 0.12 (95% CI: 0.01, 0.23)	SMD : 0.18 (95% CI: –0.42, 0.78)	SMD : 0.23 (95% CI: –0.56, 1.02)	SMD : 0.11 (95% CI: –0.27, 0.49)
Study population	11 studies, *n* = 19,969	6 studies, *n* = 4299	3 studies, *n* = 2837	10 studies, *n* = 12,833	2 studies, *n* = 12,622	1 study, *n* = 43	1 study, *n* = 25	1 study, *n* = 107
LSFF duration	12 mo–8 y	18 mo–7 y	12 mo–2 y	12 mo–8 y	4 y	2 y	2 y	2 y
CHERG score	Low	Low	Low	Low	NA	NA	NA	NA
Anemia prevalence	RR: 0.66 (95% CI: 0.59, 0.74)	RR: 0.61 (95% CI: 0.38, 0.96)	RR: 0.68 (95% CI: 0.52, 0.90)	RR: 0.66 (95% CI: 0.58, 0.76)	RR: 0.73 (95% CI: 0.64, 0.84)	—	—	—
Study population	11 studies, *n* = 140,704	7 studies, *n* = 4641	4 studies, *n* = 4092	9 studies, *n* = 131,971	3 studies, *n* = 17,063	—	—	—
LSFF duration	12 mo–16 y	12 mo–7 y	12 mo–6 y	12 mo–16 y	3–4 y	—	—	—
CHERG score	Moderate	Low	Low	Moderate	Moderate	—	—	—
Serum ferritin (μg/dL)	SMD : 0.39 (95% CI: 0.34, 0.44)	SMD : 0.47 (95% CI: 0.35, 0.59)	SMD : 0.48 (95% CI: 0.34, 0.63)	SMD : 0.36 (95% CI: 0.30, 0.42	—	—	—	—
Study population	6 studies, *n* = 6893	2 studies, *n* = 1148	1 study, *n* = 819	4 studies, *n* = 4926	—	—	—	—
LSFF duration	18 mo–8 y	7 y	6 y	18 mo–8 y	—	—	—	—
CHERG score	Moderate	NA	NA	Moderate	—	—	—	—
Iron deficiency	RR: 0.42 (95% CI: 0.32, 0.56)	RR: 0.36 (95% CI: 0.21, 0.64)	RR: 0.42 (95% CI: 0.32, 0.53)	RR: 0.46 (95% CI: 0.29, 0.72)	—	—	—	—
Study population	7 studies, *n* = 7249	3 studies, *n* = 1504	1 study, *n* = 819	4 studies, *n* = 4926	—	—	—	—
LSFF duration	18 mo–8 y	12 mo–7 y	6 y	18 mo–8 y	—	—	—	—
CHERG score	Low	Low	NA	Low	—	—	—	—

^1^Analysis model: random effects; statistical method: Mantel-Haenszel (dichotomous outcomes) and inverse-variance (continuous outcomes). CHERG, Child Health Epidemiology Research Group; Hb, hemoglobin; LSFF, large-scale food fortification; NA, not applicable; SMD, standard mean difference; WRA, women of reproductive age.

^2^Does not include pregnant women, anemic children, or anemic women.

**TABLE 6:** Summary of results for LSFF with folic acid^1^

	Age groups	
Outcome	Live + stillborn infants	WRA
Total NTD	OR: 0.59 (95% CI: 0.49, 0.70)	—
Study population	8 studies, *n* = 19,816,008	—
LSFF duration	12 mo–10 y	—
CHERG score	Moderate	—
Spina bifida	OR: 0.66 (95% CI: 0.53, 0.82)	—
Study population	9 studies, *n* = 21,175,429	—
LSFF duration (range)	12 mo–11 y	—
CHERG score	Moderate	—
Anencephaly	OR: 0.49 (95% CI: 0.40, 0.60)	—
Study population	9 studies, *n* = 21,175,429	—
LSFF duration (range)	12 mo–11 y	—
CHERG score	Moderate	—
Cephalocele	OR: 0.64 (95% CI: 0.47, 0.88)	—
Study population	8 studies, *n* = 21,049,821	—
LSFF duration	2–11 y	—
CHERG score	Moderate	—
Serum folate (nmol/L)	—	SMD: 1.25 (95% CI: 0.50, 1.99)
Study population	—	8 studies, *n* = 6765
LSFF duration (range)	—	12 mo–5 y
CHERG score	—	Low
Folate deficiency	—	RR: 0.20 (95% CI: 0.15, 0.25)
Study population	—	4 studies, *n* = 4645
LSFF duration (range)	—	12 mo–5 y
CHERG score	—	Low

^1^Analysis model: random effects; statistical method: Mantel-Haenszel (dichotomous outcomes) and inverse-variance (continuous outcomes). CHERG, Child Health Epidemiology Research Group; LSFF, large-scale food fortification; NA, not applicable; NTD, neural tube defect; SMD, standard mean difference; WRA, women of reproductive age.

## Results

**FIGURE 1:**
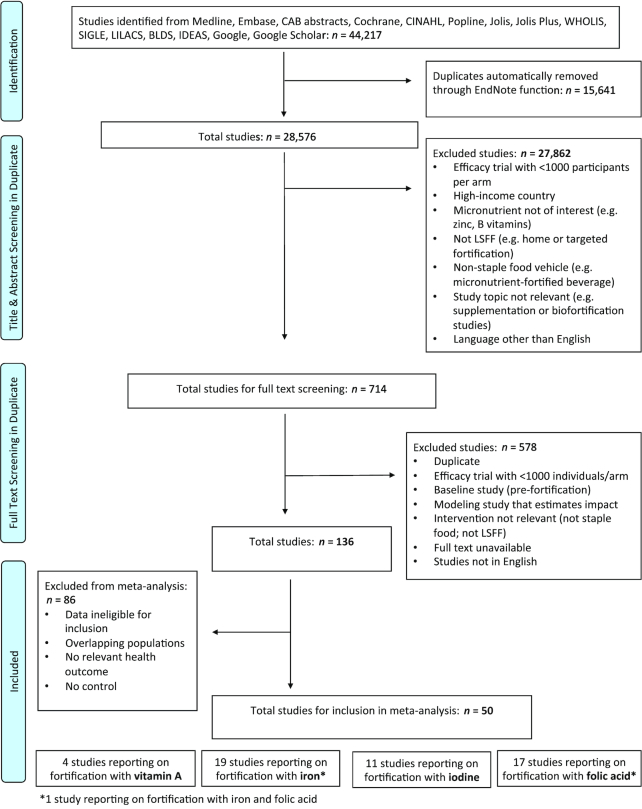
Summary of study selection.

Altogether, 136 studies fit the inclusion and exclusion criteria of the review, and 50 studies were included in the meta-analyses for vitamin A, iodine, iron, and folic acid (**Figure 1**). Complete study characteristics tables can be found in the online Supplemental Material (**Supplemental Tables 8–11**). At the screening stage, most studies were excluded because of study design (small-scale randomized trial, modeling study, or baseline study), intervention type (home or targeted fortification), intervention vehicle (not considered a staple food), study language (not published in English), or because the full text was unavailable. In total, 71 studies had to be excluded from the meta-analysis because of overlapping study populations, data that were not in usable form (e.g., no confidence limits, SD, or SE provided), outcome of interest that could not be pooled with other studies, or because there was no control estimate (whether this was a control group or a prefortification population estimate).

### Vitamin A fortification

A total of 4 studies were included in the quantitative analysis (17–20) looking at the impact of vitamin A fortification. Of these, geographic locations included Indonesia, South Africa, Guatemala, and Nicaragua, and food vehicles included sugar, maize flour, and oil. Intervention duration ranged from 12 to 24 mo, with a mean of 14 mo.

Table 3 presents a summary of results. Pooled analysis from the studies shows that vitamin A fortification is associated with a significant increase in serum retinol (SMD: 0.31; 95% CI: 0.18, 0.45) (**Supplemental Figure 1**). The effect was significant for each of the age groups assessed, and particularly for older children (SMD: 0.50; 95% CI: 0.16, 0.85). Single studies examining the effect of vitamin A fortification in children aged <1 y and WRA also signaled improvements in serum retinol.

**FIGURE 2:**
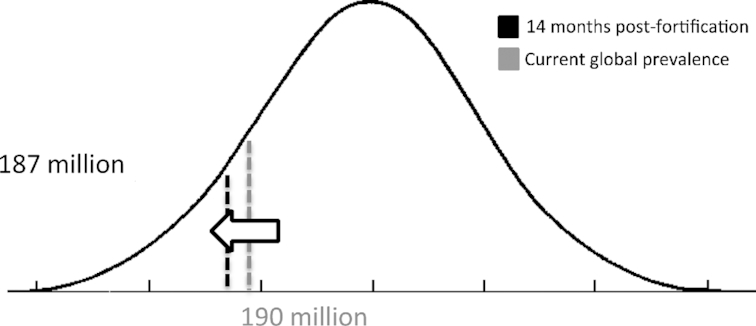
Change in distribution of global vitamin A deficiency (serum retinol <70 μmol/L) in children (0–9 y) after 14 mo of LSFF with vitamin A. LSFF, large-scale food fortification.

Considering the combined effect for all children (0–9 y), we found that serum retinol levels improved by 0.28 μg/dL (95% CI: 0.14, 0.43 μg/dL) following LSFF with vitamin A for an average of 14 mo. Today, the global prevalence of vitamin A deficiency (VAD; defined as a serum retinol concentration <0.70 μmol/L) for children aged <5 y is 33.3%, equating to 190 million children (currently, there are no global estimates for children aged 5–10 y) (21). When considering the impact of our effect estimate on a population curve, this global deficiency would shift to 32.82% (**Figure 2**), indicating an approximate reduction in VAD for 2.7 million children (95% CI: 1.3, 4.1 million children) in just over 1 y.

### Iodine fortification

Eleven studies examined the impact of salt iodization (22–33) on indicators of iodine nutritional status. Study populations consisted mostly of school-age children and adolescents (aged 5–18 y) in Asian and African locations. Iodine fortificants were varied, and included potassium iodate, sodium iodide, and multiple fortificants. Intervention duration ranged from 10 mo to 14 y, with a mean of 5.7 y.

**FIGURE 3:**
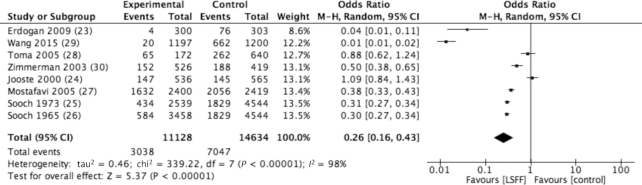
Reduction (74%) in the odds of goiter prevalence (OR: 0.26; 95% CI: 0.16, 0.43) following salt iodization. Analysis model: random effects; statistical method: Mantel-Haenszel (M-H).

A summary of results can be found in Table 4. LSFF was associated with a significant increase in urinary iodine (SMD: 1.02; 95% CI: 0.63, 1.42) (**Supplemental Figure 2**). Subgroup analysis revealed a statistically significant impact for school-age children (SMD: 1.12; 95% CI: 0.57, 1.67). Accordingly, the prevalence of iodine deficiency was reduced for this age group as well (RR: 0.25; 95% CI: 0.21, 0.29) (**Supplemental Figure 3**). Results have also demonstrated a strong decline in goiter prevalence (grade 1–2) among school-age children following fortification (OR: 0.26; 95% CI: 0.16, 0.43) (**Figure 3**), a finding that is indicative of the long-term impact of salt iodization programs.

### Iron fortification

Nineteen studies were included in the quantitative analysis (34–52) looking at the effectiveness of LSFF with iron. The study populations consisted of women and children of varying age groups, including 2 studies pertaining specifically to pregnant women and 1 looking at the effects of fortification on anemic children. The food vehicles were varied, and included maize flour, wheat flour, rice, soy sauce, fish sauce, and milk. Several fortificants were used, including sodium iron ethylenediaminetetraacetate (NaFeEDTA), ferrous sulfate, ferrous fumarate, ferrous bisglycinate, electrolytic iron, and ferric orthophosphate. In many countries, multiple iron compounds were approved for use and could differ depending on the food producer and the type of food being fortified. The studies were geographically diverse, with the majority coming from Asia and South America. One was a multicountry study, reporting on anemia prevalence in 12 different countries. Intervention duration ranged from 18 mo to 16 y, with a mean of 5.3 y.

**FIGURE 4:**
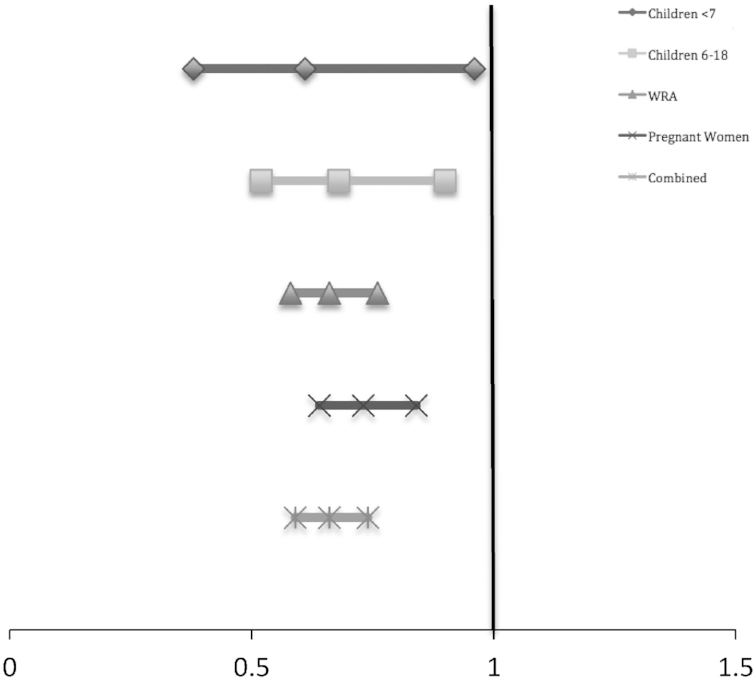
Reduction in anemia prevalence for children aged <7 y (RR: 0.61; 95% CI: 0.38, 0.96), children 6–18 y (RR: 0.68; 95% CI: 0.52, 0.90), WRA (RR: 0.66; 95% CI: 0.58, 0.76), and pregnant women (RR: 0.73; 95% CI: 0.64, 0.84), with a combined 34% reduction in anemia across all ages (RR: 0.66; 95% CI: 0.59, 0.74) following LSFF with iron. Analysis model: random effects; statistical method: Mantel-Haenszel. LSFF, large-scale food fortification; WRA, women of reproductive age.

A summary of results is presented in Table 5. Pooled analyses showed that LSFF with iron was associated with a small, but significant, increase in the hemoglobin concentration for combined populations (preschool children, school-age children, and WRA only) (**Supplemental Figure 4**). However, when disaggregating by age and status (pregnant and anemic populations at baseline) the effect remained significant for pregnant women only (SMD: 0.12; 95% CI: 0.01, 0.23). LSFF with iron was associated with a 34% decline in anemia prevalence for combined age groups (RR: 0.66; 95% CI: 0.59, 0.74) (**Figure 4** and **Supplemental Figure 5**), with the greatest impact noted for WRA (RR: 0.66; 95% CI: 0.58, 0.76), followed by school-age children (RR: 0.68; 95% CI: 0.52, 0.90) (Figure 4). There was also a statistically significant change in anemia prevalence among the youngest children (<7 y) (RR: 0.61; 95% CI: 0.38, 0.96), but this estimate generated wide CIs. Three studies looked at the impact of iron fortification in pregnant women specifically and found that, although anemia prevalence was significantly reduced (RR: 0.73; 95% CI: 0.64, 0.84), the decline was less than it was for nonpregnant women. Serum ferritin increased by 0.39 μg/L (95% CI: 0.34, 0.44 μg/L), indicating a significant improvement in iron stores for combined age groups following iron fortification (**Supplemental Figure 6**). Variability around the point estimates for serum ferritin was largest for the youngest children (SMD: 0.47; 95% CI: 0.35, 0.59). The prevalence of iron deficiency declined by 58% among all population subsets (RR: 0.42; 95% CI: 0.32, 0.56) (**Supplemental Figure 7**).

### Folic acid fortification

Folic acid fortification was identified in 17 studies (50, 51, 53–67); all but 1 (64) evaluated a national fortification program. The vast majority of studies took place in Central and South America, and food vehicles included wheat and maize flour. Intervention duration ranged from 12 mo to 11 y, with a mean of 4.2 y.

**FIGURE 5:**
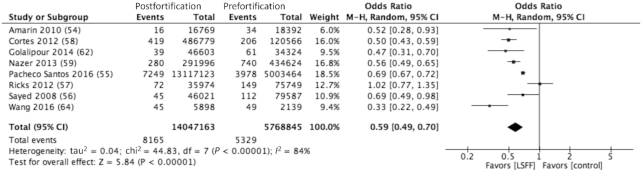
Reduction (41%) in NTD prevalence (OR: 0.59; 95% CI: 0.49, 0.70) following LSFF with folic acid. Analysis model: random effects; statistical method: Mantel-Haenszel (M-H). LSFF, large-scale food fortification; NTD, neural tube defect.

A summary of the results is presented in Table 6. Pooled analyses showed a strong association between folic acid fortification and the decreased prevalence of total NTDs (OR: 0.59; 95% CI: 0.49, 0.70) (**Figure 5**) and NTD subtype, including spina bifida (OR: 0.66; 95% CI: 0.53, 0.82) (**Supplemental Figure 8**), anencephaly (RR: 0.49; 95% CI: 0.40, 0.60) (**Supplemental Figure 9**), and cephalocele (OR: 0.64; 95% CI: 0.47, 0.88) (**Supplemental Figure 10**). A single study conducted in Peru (57) was, however, associated with an apparent increase in spina bifida following implementation of the national folic acid fortification program. Among WRA, folic acid fortification of flour was associated with a significant decline in the prevalence of folate deficiency (RR: 0.20; 95% CI: 0.15, 0.25) (**Supplemental Figure 11**), as well as improvements in serum/plasma folate levels (SMD: 1.25; 95% CI: 0.50, 1.99) (**Supplemental Figure 12**).

## Discussion

The results of this review provide evidence to support the potential of LSFF programs in LMICs. Similar to results from high-income settings, we found that LSFF had a positive impact on some functional health outcomes, including goiter, anemia, and NTD prevalence. LSFF also increased relevant micronutrient biomarker concentrations, and improved iron stores and reduced iron deficiency prevalence in both women and children. Notwithstanding the limited number of studies, we found that children aged <5 y and school-age children appeared to benefit from consumption of staple foods fortified with vitamin A in terms of serum retinol. Although we were not able to estimate direct effects on mortality, we found that a minimal (0.5%) reduction in VAD prevalence equates to ∼3 million children being protected from VAD in just over a year; an effect that, importantly, would plausibly be compounded with increasing program maturity and better intervention coverage and reach. Positive results stemming from efficacy trials could take as little as several months to be realized, whereas programs require more time to mature and achieve scale (43), given complex and variable technical, operational, and regulatory components.

We noted an age-specific effect of fortification, with a clear gradient towards higher impact among older women than among children for several outcomes examined, including serum retinol, serum ferritin, and anemia. This observation potentially relates to dietary intake of staples and micronutrient dosage, indicating that infants and younger children might not be consuming enough fortified staple foods to benefit to the same extent as mothers. To ensure adequate nutrient intakes, additional strategies should be considered for infants and young children, such as targeted fortification of complementary foods.

There have been several efforts to determine the effectiveness of iron fortification on the health status of women and children, and results have been mixed. Barkley et al. (49) found that among countries with national flour fortification initiatives (compared with those without), there was a 2.4% reduction in the odds of anemia for nonpregnant women in each year of fortification in comparison to the previous year. In contrast, Pachon et al. (68) found limited evidence to support the reduction of anemia with large-scale flour fortification. However, similar to our results, authors did note more consistent reductions in the prevalence of low ferritin.

Pre-post comparisons and other observational study designs cannot completely exclude the potential effects of confounding variables. For this and other methodologic reasons (e.g., high heterogeneity), most evidence presented here was deemed low or moderate quality. Unsurprisingly, then, systematic reviews of RCT data have provided more consistent results in terms of LSFF with iron. Three different meta-analyses demonstrated improvements in hemoglobin and serum ferritin, along with reductions in anemia prevalence following consumption of iron-fortified condiments, noodles, and other foods in LMICs (69–71). However, a review by Das et al. (15) that included pre-post designs and quasi-experimental studies along with RCTs found varying results for hemoglobin, serum ferritin, and anemia depending on the population. Because their analysis also included high-income settings, subgroup analysis by geography was possible and signaled a greater impact of fortification among populations that were deficient at baseline (15). Taken together, efficacy trials are critical to provide proof-of-concept, but they do not capture important programmatic, contextual, and individual-level factors that contribute substantially to effectiveness in real-life scenarios.

For LSFF, in particular, there are a number of factors that will influence the pathway from inception to biological impact, highlighting the complexity of this particular intervention. Aptly outlined in the WHO/CDC logic model (72), micronutrient intake and status, and ultimately function, in a target population is influenced by 3 dynamic program components: *1*) inputs (e.g., resources required), *2*) activities (e.g., policies and legislation, production and supply strategies, quality control, delivery mechanisms, communication and behavior change strategies), and *3*) outputs (e.g., access, coverage). Some of the heterogeneity in our results can be attributed to the various constituents of this framework. For example, we have noted that Peru's folic acid fortification program was unsuccessful at reducing the rate of NTDs. This may have been related to the country's legislation, which, at the time, specified a concentration of folic acid fortification below WHO recommendations (73). Several studies have demonstrated micronutrient contents in staple foods that are below recommended levels following LSFF initiation (74–78). A study based on quality assurance data from 20 national fortification programs in 12 countries has found that <50% of samples tested adhered to national fortification specifications (79). These examples highlight the need for appropriate quality-control mechanisms and adequately specified and up-to-date legal frameworks for LSFF. When interpreting our results, one must also consider coverage. The Fortification Assessment Coverage Toolkit (FACT) approach (80) was used in 2 studies that looked at multiple LSFF programs (e.g., wheat flour, maize flour, edible oil, salt) across various countries. These studies found significant variation in coverage and inequitable distribution of fortified foods that conferred a disadvantage to vulnerable population groups (81, 82). The main barriers cited were poor vehicle choice and failure to adequately fortify, underscoring issues of program planning and compliance/enforcement or insufficient consolidation of and distribution by large-scale producers, respectively (81). Interestingly, in our results we did not note any consistent pattern in effect when considering intervention duration. The exception to this was goiter prevalence, where greater declines were noted for populations that had been exposed to salt iodization for longer; a finding that highlights the utility of goiter as a long-term indicator of iodine status. Given the changing landscape of fortification programs (legislation, food vehicles, compounds, concentration, distribution methods, etc.), it is challenging to determine at what point implementation is effective at a population level. Taken together, a limitation of our study is the inability to link impact of LSFF to coverage and utilization data. Further evaluations of outcomes should be considered only where program design has been appropriate to context, and where coverage and compliance data would suggest that impact is possible and, as discussed above, where programs have been given reasonable time to mature (83).

The use of multiple strategies that overlap in terms of micronutrients delivered need to be considered in the context of excessive intakes. This is probable in countries where supplementation, fortification, and biofortification efforts exist simultaneously. There is some evidence to show excessive intake in LMICs (84–86), but generally, deficiencies are a greater problem (87). It is necessary to differentiate between a tolerable upper intake level (the level that will not pose any risk of adverse health for the general population) and toxicity, 2 terms that are not synonymous (87). Upper limits should be reviewed, especially for nutrients that pose a greater potential risk of excessive intake; with vitamin A, iodine, and iron among this group (87). In addition, there must be coordinated and defined efforts across sectors to identify all micronutrient sources (and proportion of intake by source), create common implementation strategies, and evaluate risks through ongoing monitoring and surveillance of programmatic data, food consumption, and health outcomes (87).

We have attempted to quantify the effectiveness of LSFF efforts in LMICs and, as such, have pooled estimates from a broad range of programs and voluntary population-level efforts. Although there are obvious strengths to such an analysis (e.g., improved sample), there are also limitations. We were unable to link micronutrient intake or consumption data with the outcomes examined, and therefore made an assumption that observed changes were due to fortification programs. Additionally, the majority of included studies utilized pre-post cross-sectional survey designs that limit causal inferences and increase the risk of unmeasured factors contributing to observed differences. There was significant heterogeneity between studies; differences in food vehicles, types of fortificants used, micronutrient dosage, participant age, length of study, or any other varying factors at baseline (e.g., regional differences in diet or baseline micronutrient status) made it difficult to draw overall conclusions. We aimed to perform subgroup analyses by iron fortificant, but found that the common use of unspecified or multiple iron compounds within a population did not allow for a meaningful analysis to take place. However, as there was intent to include observational studies within this analysis, heterogeneity was expected and reflects the actual differences that exist in LSFF programs globally. We used a random-effects model to account for some of these between-study differences. Similar to other reviews, we found that evaluations lacked the reporting of functional or clinical outcomes. Large-scale studies are needed to determine if there is benefit to anthropometrics, cognitive outcomes, morbidity, and mortality in WRA and children.

LSFF has now gained significant global traction as a promising approach to reduce micronutrient malnutrition, and the results of this review have provided sufficient evidence to demonstrate the positive impact of LSFF programs in LMICs. Currently, >140 countries have national salt iodization programs, 86 countries fortify ≥1 kind of cereal grain, and 40 countries mandate fortification of edible oils, ghee, or margarine (88). Although these numbers highlight important global progress, several countries that would benefit from LSFF have not yet incorporated this strategy within nutrition-related policy or programming. As a first step, countries should consider legislation of fortified grains, oil, and other staples where vulnerable populations exist and consumption of these staple foods is high. However, it will be crucial not to rely on fortification strategies alone, particularly in contexts where infection control and reduction of disease burden may play a very important role relating to program effectiveness. A coordination of the various efforts to reduce micronutrient malnutrition will be critical to ensure that micronutrient intakes are not in excess, and populations are receiving interventions that will most effectively resolve their nutritional deficits in a cost-effective manner (89). Moving forward, it will be important to consider country and regional contexts, and the various programmatic push and pull factors that will contribute to effectiveness. Although our findings are compelling, there is a need to address critical gaps in the evidence to inform LSFF program priorities, including coverage and access to fortified foods among those in greatest need. Through further research, strengthening of existing programs, and implementation of additional programs across the globe, LSFF will undoubtedly contribute to the fight against undernutrition.

## Supplementary Material

nqz023_Supplemental_File

## Notes

Supported by the Global Alliance for Improved Nutrition (GAIN).

Supplemental Tables 1–11 and Supplemental Figures 1–12 are available from the “Supplementary data” link in the online posting of the article and from the same link in the online table of contents at https://academic.oup.com/ajcn/.

Abbreviations used: CHERG, Child Health Epidemiology Research Group; LMIC, low- and middle-income country; LSFF, large-scale food fortification; NTD, neural tube defect; RCT, randomized controlled trial; SMD, standardized mean difference; VAD, vitamin A deficiency; WRA, women of reproductive age.
