# Supplementary material for: Improved micronutrient status and health outcomes in low- and middle-income countries following large-scale fortification: evidence from a systematic review and meta-analysis
Source: Am J Clin Nutr. 2019 Apr 17;109(6):1696–708. doi: 10.1093/ajcn/nqz023 (PMC6537942; doi:10.1093/ajcn/nqz023)
Supplement: nqz023_Supplemental_File [file nqz023_supplemental_file.docx]

**Improved Micronutrient Status and Health Outcome in Low and Middle Income Countries Following Large-Scale Fortification: Evidence from a Systematic Review and Meta-Analysis**

**Online Supporting Material**

**Supplemental Table 1: Data Sources.**

| **Micronutrient** | **Outcomes of Interest** |
| --- | --- |
| Databases | Medline |
|  | Embase |
|  | CAB Abstracts |
|  | Cochrane Library |
|  | Cumulative Index to Nursing and Allied Health Literature (CINAHL) |
|  | Popline |
|  | Jolis Catalogue |
|  | Jolis Plus |
|  | WHO regional databases (WHOLIS) |
|  | System for Information on Grey Literature in Europe (SIGLE) |
|  | LILACS database for Latin American and Caribbean Health Sciences Literature |
|  | British Library for Development Studies (BLDS) |
|  | IDEAS database for economics and finance research |
|  | Google and Google Scholar |

**Supplemental Table 2: Search Strategy for Medline.**

| **1. Food** |
| --- |
| exp Food/ or food*.tw. or exp Food Supply/ or exp Agricultural Crops/ or crop*.tw. or exp Flour/ or flour*.tw. or exp Salts/ or salt*.tw. or exp Fish Products/ or exp Soy Foods or sauce*.tw. or exp Cereals/ or cereal*.tw. or exp Dietary Carbohydrates/ or sugar*.tw. or exp Oryza sativa/ or rice*.tw. or exp Milk/ or milk*.tw. or exp Bread/ or bread.tw. or exp Oils/ or oil*.tw. or exp Beverages/ or beverage*.tw. or exp Yogurt/ or yogurt*.tw. or exp Margarine/ or margarine*.tw. or exp Cheese/ or cheese*.tw. or exp Zea mays/ or maize*.tw. or exp Condiments/ or condiment*.tw. or exp Triticum/ or wheat*.tw. or exp Spices/ or curry powder*.tw. or exp Dietary Fats/ or fat*.tw. or exp Dairy Products/ or dairy.tw. |
| **2. Micronutrients** |
| (exp Iron/ or iron.tw. or ferr*.tw. or exp Iodine/ or iod*.tw. or exp Vitamin A/ or "vitamin A".tw. or exp beta Carotene/ or beta carotene.tw. or exp Ferric Compounds/ or exp Folic Acid/ or folic acid.tw. or folate*.tw. or Ferrous Compounds/ or exp Micronutrients/ or micronutrient*.tw.)  **3. Fortification** |
| (exp Food, Fortified/ or fortif*.tw. or enrich*.tw.) |
| **4. Outcomes** |
| exp Deficiency Diseases/ or deficiency disease*.tw. or exp Nutrition Disorders/ or nutrition disorder*.tw. or exp Infant Nutrition Disorders/ or infant nutrition disorder*.tw. or exp Malnutrition/ or malnutrition.tw. or exp Vitamin A Deficiency/ or vitamin A deficien*.tw. or exp Folic Acid Deficiency/ or folic acid deficienc*.tw. or exp anemia/ or anemia.tw. or anemic.tw. or exp Anemia, Iron-Deficiency/ or iron-deficien* anemia.tw. or iron deficien* anemia.tw. or exp Anemia, Hypochromic/ or hypochromic anemia.tw. or micronutrient deficiency.tw. or exp Hemoglobins/ or hemoglobin*.tw. or haemoglobin*.tw.or exp Homocysteine/ or homocysteine.tw. or exp Ferritins/ or ferritin*.tw. or exp Receptors, Transferrin/ or transferrin receptors.tw. or exp Transferrin/ or exp Transferrins/ or transferrin*.tw. or exp Protoporphyrins/ or protoporphyrins.tw. or erythrocyte protophorphyrins.tw. or retinol.tw. or folate.tw. or exp Growth Disorders/ or growth disorder*.tw. or exp Wasting Syndrome/ or wasting syndrome*.tw. or wasting.tw. or stunt*.tw. or underweight.tw. or exp Thinness/ or thin*.tw. or exp Body Mass Index/ or body mass index.tw. or bmi.tw. or exp Body Height/ or body height.tw. or exp Body Weight/ or body weight.tw. or height for age.tw. or weight for age.tw. or weight for height.tw. or mid-upper arm circumference.tw. or exp Infant, Small for Gestational Age/ or small for gestational age.tw. or exp Anthropometry/ or anthropometr*.tw. or exp Body Size/ or body size.tw. or exp Congenital Abnormalities/ or congenital abnormalit*.tw. or exp Neural Tube Defects/ or neural tube defect*.tw. or NTD*.tw. or exp Spinal Dysraphism/ or spinal dysraphism.tw. or spina bifida.tw. or exp Stillbirth/ or stillbirth*.tw. or exp Congenital Hypothyroidism/ or congenital hypothyroidism.tw. or cretinism.tw. or exp Nervous System Diseases/ or nervous system disease*.tw. or neurological impairment.tw. or exp Cognition Disorders/ or cognit*.tw. or exp Mild Cognitive Impairment/ or cognitive impairment.tw. or exp Night Blindness/ or night blindness.tw. or exp Xerophthalmia/ or xerophthalm*.tw. or exp Hypothyroidism/ or hypothyroid*.tw. or exp Goiter/ or (goiter or goitre).tw. or exp Diarrhea/ or exp Diarrhea, Infantile/ or diarrhea.tw. or exp Pneumonia/ or pneumonia.tw. or exp Malaria/ or malaria.tw. or exp Urinary Tract Infections/ or urinary tract infection*.tw. or UTI.tw. or exp Fever/ or fever*.tw. or exp Morbidity/ or morbidity.tw. or exp Fetal Mortality/ or exp Maternal Mortality/ or exp Child Mortality/ or exp Infant Mortality/ or exp Mortality/ or mortality.tw. or exp Heart Diseases/ or heart disease*.tw. or exp Stroke/ or stroke.tw. or exp Depression/ or depression.tw |
| **5. Population** |
| ("developing country" OR "developing countries" OR "developing nation" OR "developing nations" OR "developing population" OR "developing populations" OR "developing world" OR "less developed country" OR "less developed countries" OR "less developed nation" OR "less developed nations" OR "less developed population" OR "less developed populations" OR "less developed world" OR "lesser developed country" OR "lesser developed countries" OR "lesser developed nation" OR "lesser developed nations" OR "lesser developed population" OR "lesser developed populations" OR "lesser developed world" OR "under developed country" OR "under developed countries" OR "under developed nation" OR "under developed nations" OR "under developed population" OR "under developed populations" OR "under developed world" OR "underdeveloped country" OR "underdeveloped countries" OR "underdeveloped nation" OR "underdeveloped nations" OR "underdeveloped population" OR "underdeveloped populations" OR "underdeveloped world" OR "middle income country" OR "middle income countries" OR "middle income nation" OR "middle income nations" OR "middle income population" OR "middle income populations" OR "low income country" OR "low income countries" OR "low income nation" OR "low income nations" OR "low income population" OR "low income populations" OR "lower income country" OR "lower income countries" OR "lower income nation" OR "lower income nations" OR "lower income population" OR "lower income populations" OR "underserved country" OR "underserved countries" OR "underserved nation" OR "underserved nations" OR "underserved population" OR "underserved populations" OR "underserved world" OR "under served country" OR "under served countries" OR "under served nation" OR "under served nations" OR "under served population" OR "under served populations" OR "under served world" OR "deprived country" OR "deprived countries" OR "deprived nation" OR "deprived nations" OR "deprived population" OR "deprived populations" OR "deprived world" OR "poor country" OR "poor countries" OR "poor nation" OR "poor nations" OR "poor population" OR "poor populations" OR "poor world" OR "poorer country" OR "poorer countries" OR "poorer nation" OR "poorer nations" OR "poorer population" OR "poorer populations" OR "poorer world" OR "developing economy" OR "developing economies" OR "less developed economy" OR "less developed economies" OR "lesser developed economy" OR "lesser developed economies" OR "under developed economy" OR "under developed economies" OR "underdeveloped economy" OR "underdeveloped economies" OR "middle income economy" OR "middle income economies" OR "low income economy" OR "low income economies" OR "lower income economy" OR "lower income economies" OR "low gdp" OR "low gnp" OR "low gross domestic" OR "low gross national" OR "lower gdp" OR "lower gnp" OR "lower gross domestic" OR "lower gross national" OR lmic OR lmics OR "third world" OR "lami country" OR "lami countries" OR "transitional country" OR "transitional countries" OR Africa OR Asia OR Caribbean OR West Indies OR South America OR Latin America OR Central America OR Afghanistan OR Albania OR Algeria OR Angola OR Antigua OR Barbuda OR Argentina OR Armenia OR Armenian OR Aruba OR Azerbaijan OR Bangladesh OR Benin OR Byelarus OR Byelorussian OR Belarus OR Belorussian OR Belorussia OR Belize OR Bhutan OR Bolivia OR Bosnia OR Herzegovina OR Hercegovina OR Botswana OR Brazil OR Bulgaria OR Burkina Faso OR Burkina Fasso OR Upper Volta OR Burundi OR Urundi OR Cambodia OR Khmer Republic OR Kampuchea OR Cameroon OR Cameroons OR Cameron OR Camerons OR Cape Verde OR Central African Republic OR Chad OR Chile OR China OR Colombia OR Comoros OR Comoro Islands OR Comores OR Mayotte OR Congo OR Zaire OR Costa Rica OR Cote d'Ivoire OR Ivory Coast OR Cuba OR Cyprus OR Czechoslovakia OR Czech Republic OR Slovakia OR Slovak Republic OR Djibouti OR French Somaliland OR Dominica OR Dominican Republic OR East Timor OR East Timur OR Timor Leste OR Ecuador OR Egypt OR United Arab Republic OR El Salvador OR Eritrea OR Estonia OR Ethiopia OR Fiji OR Gabon OR Gabonese Republic OR Gambia OR Gaza OR Georgia Republic OR Georgian Republic OR Ghana OR Gold Coast OR Greece OR Grenada OR Guatemala OR Guinea OR Guam OR Guiana OR Guyana OR Haiti OR Honduras OR India OR Maldives OR Indonesia OR Iran OR Iraq OR Isle of Man OR Jamaica OR Jordan OR Kazakhstan OR Kazakh OR Kenya OR Kiribati OR Korea OR Kosovo OR Kyrgyzstan OR Kirghizia OR Kyrgyz Republic OR Kirghiz OR Kirgizstan OR "Lao PDR" OR Laos OR Latvia OR Lebanon OR Lesotho OR Basutoland OR Liberia OR Libya OR Lithuania OR Macedonia OR Madagascar OR Malagasy Republic OR Malaysia OR Malaya OR Malay OR Sabah OR Sarawak OR Malawi OR Nyasaland OR Mali OR Malta OR Marshall Islands OR Mauritania OR Mauritius OR Agalega Islands OR Mexico OR Micronesia OR Middle East OR Moldova OR Moldovia OR Moldovian OR Mongolia OR Montenegro OR Morocco OR Ifni OR Mozambique OR Myanmar OR Myanma OR Burma OR Namibia OR Nepal OR Netherlands Antilles OR New Caledonia OR Nicaragua OR Niger OR Nigeria OR Northern Mariana Islands OR Muscat OR Pakistan OR Palau OR Palestine OR Panama OR Paraguay OR Peru OR Philippines OR Philipines OR Phillipines OR PhillippinesOR Puerto Rico OR Romania OR Rumania OR Roumania OR Russia OR Russian OR Rwanda OR Ruanda OR Saint Kitts OR St Kitts OR Nevis OR Saint Lucia OR St Lucia OR Saint Vincent OR St Vincent OR Grenadines OR Samoa OR Samoan Islands OR Navigator Island OR Navigator Islands OR Sao Tome OR Senegal OR Serbia OR Montenegro OR Seychelles OR Sierra Leone OR Slovenia OR Sri Lanka OR Ceylon OR Solomon Islands OR Somalia OR Sudan OR Suriname OR Surinam OR Swaziland OR Syria OR Tajikistan OR Tadzhikistan OR Tadjikistan OR Tadzhik OR Tanzania OR Thailand OR Togo OR Togolese Republic OR Tonga OR Tunisia OR Turkey OR Turkmenistan OR Turkmen OR Uganda OR Ukraine OR Uruguay OR USSR OR Soviet Union OR Union of Soviet Socialist Republics OR Uzbekistan OR Uzbek OR Vanuatu OR New Hebrides OR Venezuela OR Vietnam OR Viet Nam OR West Bank OR Yemen OR Yugoslavia OR Zambia OR Zimbabwe OR Rhodesia).tw. |
| 1. 2 and 3 2. 3 and 4 3. 1 and 3 4. 1 and 2 and 3 5. 1 and 2 and 3 and 4 6. OR/ A-E 7. F and 5 |

**Supplemental Table 3: Outcomes analyzed per micronutrient.**

| **Micronutrient** | **Outcomes of Interest** |
| --- | --- |
| Vitamin A | Serum retinol, haemoglobin concentration |
| Iron | Anemia prevalence, haemoglobin concentration, iron-deficiency anemia, iron-deficiency, serum ferritin |
| Iodine | Mean urinary iodine, iodine deficiency, goiter prevalence |
| Folic acid | Neural tube defects, including spina bifida, anencephaly, cephalocele, serum/plasma folate, folate deficiency |

**Supplemental Table 4: Quality assessment for studies of vitamin A.**

| Quality assessment | | | | | | Summary of findings | | | | **Overall Quality of Evidence** | |
| --- | --- | --- | --- | --- | --- | --- | --- | --- | --- | --- | --- |
| **Number of Studies** | **Design** | | **Study Quality** | **Consistency** | **Sub-group** | **Number of participants in intervention group** | **Number of participants in control group** | **Effect size (95% CI)** | **P-value** |  |  |
| OUTCOME: Serum retinol | | | | | | | | | | | |
| 4 | | RCT/cohort/before-after/ repeated cross-sectional | Quality ranges from very low to moderate | Heterogeneity (I²=58%) | **Combined effect** | 1319 | 1481 | 0.31  [0.18, 0.45] | <0.00001 | | **Moderate** |
| 1 | Repeated cross-sectional | | Single study of moderate quality | Heterogeneity (N/A) | Children <12 months of age | 356 | 343 | 0.31 [0.16, 0.46] | <0.00001 | | N/A |
| 3 | | RCT/ before-after/ repeated cross-sectional | Quality ranges from very low to moderate | Heterogeneity (I²=0%) | Children 12-59 months of age | 580 | 583 | 0.14 [0.02, 0.25] | 0.02 | | Moderate |
| 2 | Cohort | | Quality ranges from low to moderate | Heterogeneity (I²=35%) | Children 5-9 years of age | 210 | 281 | 0.50 [0.16, 0.85] | 0.004 | | Low |
| 1 Single study of moderate quality | Cohort | | Single study of moderate quality | Heterogeneity (N/A) | WRA | 171 | 171 | 0.45 [0.26, 0.64] | <0.00001 | | N/A |

**Supplemental Table 5: Quality assessment for studies of iodine.**

| Quality assessment | | | | | | Summary of findings | | | | **Overall Quality of Evidence** |
| --- | --- | --- | --- | --- | --- | --- | --- | --- | --- | --- |
| **Number of Studies** | **Design** | | **Study Quality** | **Consistency** | **Sub-group** | **Number of participants or events in intervention group^1^** | **Number of participants or events in control group^1^** | **Effect size (95% CI)** | **P-value** |  |
| OUTCOME: Urinary iodine concentration | | | | | | | | SMD |  |  |
| 4 | | RCT/cohort/cross-sectional | Quality ranges from low to moderate | Heterogeneity (I^2^=98%) | **Combined effect** | 3830 | 5033 | 1.02  (0.63, 1.42) | <0.00001 | **Low** |
| 4 | RCT/cohort/cross-sectional | | Quality ranges from low to moderate | Heterogeneity (I^2^=99%) | SAC (5-18 years) | 2370 | 4501 | 1.12  (0.57, 1.67) | <0.0001 | Low |
| 1 | | Cross-sectional | Single study of low quality | Heterogeneity (N/A) | WRA | 1460 | 532 | 0.65 (0.54, 0.75) | <0.00001 | N/A |
| OUTCOME: Iodine deficiency | | | | | | | | Risk Ratio |  |  |
| 3 | RCT/cross-sectional | | All low quality studies | Heterogeneity (I^2^=0%) | **Combined effect** | 148 | 651 | 0.25  (0.21, 0.29) | <0.00001 | **Low** |
| 3 | RCT/cross-sectional | | All low quality studies | Heterogeneity (I^2^=0%) | SAC | 137 | 608 | 0.25  (0.21, 0.29) | <0.00001 | Low |
| 1 | RCT | | Single study of low quality | Heterogeneity (I^2^=0%) | WRA | 11 | 43 | 0.26  (0.15, 0.45) | <0.00001 | N/A |
| OUTCOME: Goiter | | | | | | | | Odds Ratio |  |  |
| 8 | cRCT/cohort/cross-sectional | | Quality ranges from very low to moderate | Heterogeneity (I^2^=98%) | **Combined effect for SAC** | 3038 | 7047 | 0.26  (0.16, 0.43) | <0.00001 | **Moderate** |

^1^Number of events reported for dichotomous outcomes and number of participants reported for continuous outcomes.

**Supplemental Table 6: Quality assessment for studies of iron.**

| Quality assessment | | | | | | Summary of findings | | | | **Overall Quality of Evidence** |
| --- | --- | --- | --- | --- | --- | --- | --- | --- | --- | --- |
| **Number of Studies** | **Design** | | **Study Quality** | **Consistency** | **Sub-group** | **Number of participants or events in intervention group^1^** | **Number of participants or events in control group^1^** | **Effect size (95% CI)** | **P-value** |  |
| OUTCOME: Hemoglobin concentration | | | | | | | | SMD |  |  |
| 11 | | RCT/cRCT/ cohort/ before-after/cross-sectional | Quality ranges from very low to high | Heterogeneity (I² = 97%) | **Combined effect^2^** | 10,418 | 9551 | 0.19  (0.04, 0.35) | 0.02 | **Low** |
| 6 | RCT/before-after/cross-sectional | | Quality ranges from very low to high | Heterogeneity (I² = 96%) | Children <7 years | 2111 | 2188 | 0.30  (-0.05, 0.66) | 0.09 | Low |
| 3 | | RCT/cohort | Quality ranges from very low to high | Heterogeneity (I² = 86%) | Children 6-18 years | 1466 | 1371 | 0.13  (-0.08, 0.35) | 0.23 | Low |
| 10 | RCT/cRCT/ cohort/ before-after/cross-sectional | | Quality ranges from very low to high | Heterogeneity (I² = 97%) | WRA | 6841 | 5992 | 0.15  (-0.07, 0.37) | 0.18 | Low |
| 2 | Cross-sectional | | 1 study of low quality and 1 study of moderate quality | Heterogeneity (I² = 88%) | Pregnant women | 5954 | 6668 | 0.12  (0.01, 0.23) | 0.03 | Low |
| 1 | RCT | | Single study of very low quality | Heterogeneity (N/A) | Anemic children 1-5 years | 22 | 21 | 0.18  (-0.42, 0.78) | 0.55 | N/A |
| 1 | RCT | | Single study of very low quality | Heterogeneity (N/A) | Anemic children 5-10 years | 12 | 13 | 0.23  (-0.56, 1.02) | 0.57 | N/A |
| 1 | RCT | | Single study of very low quality | Heterogeneity (N/A) | Anemic WRA | 49 | 58 | 0.11  (-0.27, 0.49) | 0.56 | N/A |
| OUTCOME: Anemia | | | | | | | | Risk Ratio |  |  |
| 11 | RCT/cRCT/  cohort/time series/cross-sectional | | Quality ranges from very low to high | Heterogeneity (I² = 96%) | **Combined effect^2^** | 20,501 | 14,404 | 0.66  (0.59, 0.74) | <0.00001 | **Moderate** |
| 7 | RCT/cRCT/ cross-sectional | | Quality ranges from low to high | Heterogeneity (I² = 93%) | Children <7 years | 565 | 587 | 0.61  (0.38, 0.96) | 0.03 | Low |
| 4 | RCT/cohort/ cross-sectional | | Quality ranges from very low to high | Heterogeneity (I² = 71%) | Children 6-18 years | 323 | 477 | 0.68  (0.52, 0.90) | 0.006 | Low |
| 9 (1 multi-country study) | RCT/cRCT/ cohort/cross-sectional | | Quality ranges from very low to high | Heterogeneity (I² = 97%) | WRA | 19,613 | 13,340 | 0.66  (0.58, 0.76) | <0.00001 | Moderate |
| 3 | Cross-sectional | | 1 study of low quality and 2 studies of moderate quality | Heterogeneity (I² = 87%) | Pregnant women | 2448 | 3959 | 0.73  (0.64, 0.84) | <0.00001 | Moderate |
| OUTCOME: Serum ferritin | | | | | | | | SMD |  |  |
| 6 | cRCT/before-after/cross-sectional | | Quality ranges from low to moderate | Heterogeneity (I² = 67%) | **Combined effect** | 3781 | 3112 | 0.39  (0.34, 0.44) | <0.00001 | **Moderate** |
| 2 | Cross-sectional | | 2 studies of moderate quality | Heterogeneity (I² = 65%) | Children 1-7 years | 667 | 481 | 0.47  (0.35, 0.59) | <0.00001 | Moderate |
| 1 | Cross-sectional | | Single study of low quality | Heterogeneity (N/A) | Children 7-15 years | 537 | 282 | 0.48  (0.34, 0.63) | <0.00001 | N/A |
| 4 | cRCT/before-after/cross-sectional | | 1 study of low quality and 3 studies of moderate quality | Heterogeneity (I² = 72%) | WRA | 2577 | 2349 | 0.36  (0.30, 0.42) | <0.00001 | Moderate |
| OUTCOME: Iron deficiency | | | | | | | | Risk Ratio |  |  |
| 7 | cRCT/before-after/cross-sectional | | Quality ranges from low to moderate | Heterogeneity (I² = 84%) | **Combined effect** | 486 | 812 | 0.42  (0.32, 0.56) | <0.00001 | **Low** |
| 3 | cRCT/cross-sectional | | 3 studies of moderate quality | Heterogeneity (I² = 78%) | Children 1-7 years | 85 | 149 | 0.36  (0.21, 0.64) | <0.00001 | Low |
| 1 | Cross-sectional | | Single study of low quality | Heterogeneity (N/A) | Children 7-15 years | 83 | 105 | 0.42  (0.32, 0.53) | <0.00001 | N/A |
| 4 | cRCT/before-after/cross-sectional | | 1 study of low quality and 3 studies of moderate quality | Heterogeneity (I² = 89%) | WRA | 318 | 558 | 0.46  (0.29, 0.72) | 0.0008 | Low |

^1^Number of events reported for dichotomous outcomes and number of participants reported for continuous outcomes. ^2^Combined effect does not include pregnant women, anemic children, or anemic women of reproductive age.

**Supplemental Table 7: Quality assessment for studies of folic acid.**

| Quality assessment | | | | | | | | Summary of findings | | | | **Overall Quality of Evidence** |
| --- | --- | --- | --- | --- | --- | --- | --- | --- | --- | --- | --- | --- |
| **Number of Studies** | **Design** | | **Study Quality** | **Consistency** | | **Sub-group** | | **Number of participants or events in intervention groups^1^** | **Number of participantsor events in control group^1^** | **Effect size (95% CI)** | **P-value** |  |
| OUTCOME: NTD | | | | | | | | | | Odds Ratio |  |  |
| 8 | | RCT/hospital-based records/ hospital-based surveillance | Quality ranges from low to moderate | | Heterogeneity (I^2^=84%) | | **Combined effect for infants (live and stillborn)** | 8165 | 5329 | 0.59  (0.49, 0.70) | <0.00001 | **Moderate** |
| OUTCOME: Spina bifida | | | | | | | | | | Odds Ratio |  |  |
| 9 | RCT/ hospital-based records/ hospital-based surveillance | | Quality ranges from low to moderate | Heterogeneity (I^2^=88%) | | **Combined effect for infants (live and stillborn)** | | 3820 | 2565 | 0.66  (0.53, 0.82) | 0.0002 | **Moderate** |
| OUTCOME: Anencephaly | | | | | | | | | | Odds Ratio |  |  |
| 9 | RCT/ hospital-based records/ hospital-based surveillance | | Quality ranges from low to moderate | Heterogeneity (I^2^=78%) | | **Combined effect for infants (live and stillborn)** | | 3907 | 2969 | 0.49  (0.40, 0.60) | <0.00001 | **Moderate** |
| OUTCOME: Cephalocele | | | | | | | | | | Odds Ratio |  |  |
| 8 | RCT/ hospital-based records/ hospital-based surveillance | | Quality ranges from low to moderate | Heterogeneity (I^2^=75%) | | **Combined effect for infants (live and stillborn)** | | 1181 | 676 | 0.64  (0.47, 0.88) | 0.006 | **Moderate** |
| OUTCOME: Folate deficiency | | | | | | | | | | Risk Ratio |  |  |
| 4 | Propsective cohort/ cross-sectional | | 1 study of very low quality, 2 studies of low quality, and 1 study of moderate quality | Heterogeneity (I^2^=35%) | | **Combined effect for WRA** | | 68 | 429 | 0.20 (0.15, 0.25) | <0.00001 | **Low** |
| OUTCOME: Serum folate | | | | | | | | | | SMD |  |  |
| 8 | RCT/  prospective cohort/ before-after/ cross-sectional | | Quality ranges from very low to moderate | Heterogeneity (I^2^=99%) | | **Combined effect for WRA** | | 3325 | 3440 | 1.25 (0.50, 1.99) | 0.001 | **Low** |

^1^Number of events reported for dichotomous outcomes and number of participants reported for continuous outcomes.

**Supplemental Table 8: Study characteristics table for vitamin A studies.**

| **Vitamin A** | | | | | | | | | |
| --- | --- | --- | --- | --- | --- | --- | --- | --- | --- |
| Author | Publication Year | Country | Year of LSFF Implementation | Mandatory/  Voluntary | Study Design | Target Population | Fortification Vehicle | Fortificant Compound | Concentration of Fortificant |
| Arroyave [1] | 1981 | Guatemala | 1975 | Mandatory | Before-after | Children aged 1-5 years | Sugar | Retinyl palmitate | 10μg/g |
| Nesamvuni [2] | 2005 | South Africa | NA | Mandatory | RCT | Children between 1-3 years of age at a local health centre who were below the 5th percentile of weight for age or height in the specified age range | Maize flour | Not specified | 1700IU/150g |
| Ribaya-Mercado [3] | 2004 | Nicaragua | 1999 | Mandatory | Observational cohort | School aged children 5-9 years of age | Sugar | Not specified | 10 – 15μg retinol activity equivalents (RAE)/g [1μg retinol = 1 RAE] |
| Sandjaja [4] | 2014 | Indonesia | 2013 | Mandatory | Repeated cross-sectional or cohort (depending on population subgroup) | Infants 12-59 months, children 5-9 years of age, and WRA sampled from poor households | Oil | Retinyl palmitate | 13.6mg/kg |

**Supplemental Table 9: Study characteristics table for iodine studies.**

| **Iodine** | | | | | | | | | |
| --- | --- | --- | --- | --- | --- | --- | --- | --- | --- |
| Author | Publication Year | Country | Year of LSFF Implementation | Mandatory/  Voluntary | Study Design | Study Population | Fortification Vehicle | Fortificant Compound | Concentration of Fortificant |
| Andersson [5] | 2008 | India | Not specified | Not specified | RCT | 5-15 year old children | Salt | Potassium iodate | 30 μg/g |
| Asibey-Berko [6] | 2007 | Ghana | 1920 | Not specified | RCT | Mildly anemic and non-anemic, healthy, non-pregnant women aged 15-45 years and their children aged 1-5 years | Salt | Potassium iodide | 50 mg/kg |
| Erdogan [7] | 2009 | Turkey | 1999 | Mandatory | Repeated cross-sectional | School children aged 9-11 years | Salt | Potassium iodide or potassium iodate | Potassium iodide: 50-70mg/kg Potassium iodate: 25-40 mg/kg |
| Jooste [8] | 2000 | South Africa | 1995 | Mandatory | Repeated cross-sectional | School children in grades 4-7 in four communities of Haarlem, Louterwater, Krakeel, and Joubertina | Salt | Potassium iodate | 40-60 μg/g |
| Mostafavi [9] | 2005 | Iran | 1989 | Mandatory | Repeated cross-sectional | Randomly selected students (in previously selected schools) aged 6-18 years in city of Shiraz | Salt | Not specified | Not specified |
| NNS [10, 11] | 2001/ 2011 | Pakistan | Not specified | Mandatory | Repeated cross-sectional | WRA and school aged children sampled for the 2001 and 2011 national nutrition surveys | Salt | Not specified | Not specified |
| Sooch [12] | 1965 | India | 1957 | Voluntary | cRCT | School-aged children in zones A and C who received fortified salt and in zone B (control) | Salt | Potassium iodide and potassium iodate | Potassium iodide: 1/50,000 parts salt  potassium iodate: 1/40,000 parts salt |
| Sooch [13] | 1973 | India | 1962 | Voluntary | Cohort | Children attending schools in zones A, B, and C who were previously surveyed | Salt | Potassium iodate | 1/40,000 parts salt |
| Toma [14] | 2005 | Romania | 2002 | Mandatory | Repeated cross-sectional | School children aged 6-14 years | Salt and iodized bread | Iodine or potassium iodate | Iodine: 20mg/kg potassium iodate: 34 ± 8.5mg/kg |
| Wang [15] | 2015 | China | 1996 | Mandatory | Repeated cross-sectional | School children aged 8-10 years | Salt | Not specified | Not specified |
| Zimmermann [16] | 2003 | Cote d’Ivoire | 1998 | Mandatory | Time series | School children aged 5-14 years | Salt | Not specified | Not specified |

**Supplemental Table 10: Study characteristics table for iron studies.**

| **Iron** | | | | | | | | | |
| --- | --- | --- | --- | --- | --- | --- | --- | --- | --- |
| Author | Publication Year | Country | Year of LSFF Implementation | Mandatory/  Voluntary | Study Design | Study Population | Fortification Vehicle | Fortificant Compound | Concentration of Fortificant |
| Angeles-Agdeppa [17] | 2011 | Philippines | Not specified | Mandatory | Cohort | Mother—child pairs (6-9 years of age) | Rice | Micronized dispersible ferric pyrophosphate (MDFP) | 600mg/100g |
| Anonymous [18] | 1982 | India | NA | Voluntary | RCT | Children 7-18 years of age, WRA | Salt | Ferric orthophosphate, iron sulphate | Not specified |
| Assuncao [19] | 2007 | Brazil | 2004 | Mandatory | Repeated cross-sectional | Children aged 0-71 months | Flour | NaFeEDTA, Electrolytic Iron, Dehydrated Iron Sulphate, Iron Fumerate, Reduced Iron, Chelated Iron Bysglicin | 4.2mg/100g |
| Assuncao [20] | 2012 | Brazil | 2004 | Mandatory | Repeated cross-sectional | Children 2-6 years of age | Wheat flour and maize flour | NaFeEDTA, Ferrous sulfate, ferrous fumerate, reduced iron, iron bisglycinate, electrolytic iron | 4.2mg/100g |
| Barkley [21] | 2015 | Bolivia, Costa Rica, Fiji, Honduras, Indonesia, Jordan, Mexico, Nicaragua, Peru, Philippines, Senegal, Uzbekistan | Bolivia 1998; Costa Rica 1997; Fiji 2005; Honduras 1997; Indonesia 2001; Jordan 2002; Mexico 1996; Nicaragua 1997; Peru 1997; Philippines 2004; Senegal 2009; Uzbekistan 2005 | Mandatory | Repeated cross-sectional | Countries were included if there were at least 2 nationally representative anemia surveys conducted on non-pregnant women (one prior to flour fortification and one following) | Wheat flour (wheat and maize flour for Costa Rica and Mexico) | Not specified | Not specified |
| Chakrabarti [22] | 2018 | India | 2008 | Mandatory (within Public Distribution System) | Repeated cross-sectional | Pregnant women (15-45 years) in Punjab and Tamil Nadu (intervention states) and Haryana, Andhra Pradesh, Kamataka, and Kerala (control states) | Wheat flour | Not specified | 60mg/kg (Tamil Nadu) and 30mg/kg (Punjab) |
| Chen [23] | 2005 | China | 2000 | Voluntary | RCT | Subjects (>3 years) given iron-fortified soy sauce | Soy sauce | NaFeEDTA | 29.6mg/100ml |
| da Silva [24] | 2012 | Brazil | 2004 | Mandatory | Repeated cross-sectional | Pregnant women with date of last menstrual period after June 2005 who attended one of two public maternity hospitals in Rio de Janeiro | Wheat flour and maize flour | Ferrous sulfate (dehydrated), ferrous fumarate, reduced Fe-325 mesh Tyler, electrolytic Fe-325 mesh Tyler, NaFeEDTA, or Fe bis-glycine chelate | 4.2mg/100g |
| Engle-Stone [25] | 2017 | Cameroon | 2011 | Mandatory | Repeated cross-sectional | Eligible households had at least 1 child aged 12-59 months and 1 woman of reproductive age (15-49 years) who was the child’s primary caregiver | Wheat flour | Ferrrous fumarate | 60mg/kg |
| Fujimori [26] | 2011 | Brazil | 2004 | Mandatory | Repeated cross-sectional | Pregnant women with date of last menstrual period after June 2005 | Wheat flour and maize flour | Not specified | 4.2mg/100g |
| Kalimbira [27] | 2010 | Malawi | 1996 | Voluntary | Repeated cross-sectional | Non-pregnant women aged 15-49 years living in MICAH areas that participated in the program throughout its course | Maize flour or maize flour blended with legumes | Not specified | Not specified |
| Layrisse [28] | 2002 | Venezuela | 1993 | Mandatory | Repeated cross-sectional | Children from 7, 11, and 15 years of age from low SES | Maize flour and wheat flour | Ferrous fumarate | Maize flour: 50mg/kg  Wheat flour: 20mg/kg  * Due to a problem with change in the colour of the maize bread, from 1994 the maize flour was fortified with 30mg/kg of ferrous fumarate and 20mg of electrolytic iron |
| Martorell [29] | 2015 | Costa Rica | 1958 (reduced iron in wheat flour), amended in 2002 (ferrous fumarate); 1999 (ferrous bisglycinate to maize flour) | Mandatory | Repeated cross-sectional | Pregnant & non-pregnant women aged 15-45 (those breastfeeding a child <6mo of age excluded) and  children aged 1-7 years | Wheat flour, maize flour and milk | Ferrous fumarate or ferrous bisglycinate | Wheat flour:  55mg/kg  Maize flour: 22mg/kg  Milk: 1.4mg/250mL |
| National Food and Nutrition Centre [30] | 2012 | Fiji | 2005 | Not specified | Repeated cross-sectional | Non-pregnant women aged 15-45 years randomly selected and matched for age, ethnicity, and Enumeration Areas with the 2004 Micronutrient Survey | Flour | Elemental iron powder | 60 mg/kg |
| Nestel [31] | 2004 | Sri Lanka | 1998 | Voluntary | RCT | Preschool children (9 to 71 months), primary school children (6 to 10.9 years) or WRA; Exclusion of pregnant women and people with severe anemia | Flour | Electrolytic Iron  and reduced Iron | Electrolytic Iron: 78±15.2 mg/kg,  Reduced iron: 76±6.6 mg/kg,  Unfortified flour: 15±1.7 |
| Rivera [32] | 2010 | Mexico | 2002 | Not specified | RCT | Infants aged 12-30 months | Milk (powdered form) | Ferrous gluconate | 5.28 mg (in 48 g powder or 400 mL reconstituted milk) |
| Sadighi [33] | 2009 | Iran | 2001 | Mandatory | Before-after | WRA receiving fortified wheat flour in Golestan province | Wheat flour and bread | Ferrous sulphate | Not specified |
| Van Thuy [34] | 2005 | Vietnam | 2001 | Voluntary | cRCT | Women aged 16 to 49 from villages with anemia prevalence >20% | Fish sauce | NaFeEDTA | 9mmol/L |
| Wang [35] | 2009 | China | 2004 | Voluntary | Repeated cross-sectional | Non-pregnant women ≥20 years and children aged 3 to 7 years | Soy sauce | NaFeEDTA | 25mg/100ml |

**Supplemental Table 11: Study characteristics table for folic acid studies.**

| **Folic Acid** | | | | | | | | | |
| --- | --- | --- | --- | --- | --- | --- | --- | --- | --- |
| Author | Publication Year | Country | Year of LSFF Implementation | Mandatory/  Voluntary | Study Design | Study Population | Fortification Vehicle | Fortificant  Compound | Concentration of Fortificant |
| Abdollahi [36] | 2011 | Iran | 2006 | Mandatory | Prospective hospital-based surveillance and repeated cross-sectional | Non-pregnant WRA with no history of chronic disease | Wheat flour | Folic acid | 1.5mg/kg |
| Amarin [37] | 2010 | Jordan | 2002 | Mandatory | Retrospective hospital-based records | All births occurring at the Princess Badea Teaching Hospital | Flour | Folic acid | 1.5mg/kg |
| Chen [38] | 2004 | Costa Rica | 1998 | Mandatory | Repeated cross-sectional | Non-pregnant, non-lactating women 15-44 years of age | Wheat flour and maize flour | Folic Acid | Wheat Flour: 1.5mg/kg and 1.8mg/kg  maize Flour: 1.3mg/kg and 1.8mg/kg |
| Cortes [39] | 2012 | Chile | 2000 | Mandatory | Prospective hospital-based surveillance | All live and still birth infants weighing at least 500g born in 9 hospitals in the capital of Chile between 2001-2009 | Wheat flour | Folic acid | 2.2mg/kg |
| Engle-Stone [25] | 2017 | Cameroon | 2011 | Mandatory | Repeated cross-sectional | Eligible households had at least 1 child aged 12-59 months and 1 woman of reproductive age (15-49 years) who was the child’s primary caregiver | Wheat flour | Folic acid | 5.0mg/kg |
| Golalipour [40] | 2014 | Iran | 2007 | Mandatory | Prospective hospital-based surveillance | All live births and therapeutic abortions in 16 hospitals in Golestan province from March 2006 to Sept 2009 | Wheat flour | Folic acid | 1.5mg/kg |
| Hertrampf [41] | 2008 | Chile | 2000 | Mandatory | Before-after | All live and still birth infants weighing at least 500g born in 9 hospitals in the capital of Chile between 2001-2009 and WRA who have 1 child and no family history of disease | Wheat flour | Folic acid | 2.2mg/kg |
| Lopez-Camelo [42] | 2010 | Chile, Argentina, Brazil | Chile: 2000, Argentina: 2003, Brazil: 2004 | Mandatory | Retrospective hospital-based records | Live and stillborn infants weighing > 500g | Wheat flour and maize flour | Folic acid | Chile: 2.2mg/kg, Argentina: 2.2mg/kg, Brazil: 1.5mg/kg |
| National Food and Nutrition Centre [30] | 2012 | Fiji | 2005 | Not specified | Repeated cross-sectional | Non-pregnant women aged 15-45 years randomly selected and matched for age, ethnicity, and Enumeration Areas with the 2004 Micronutrient Survey | Flour | Folic acid | 1.5 mg/kg |
| Nazer [43] | 2013 | Chile | 2000 | Mandatory | Retrospective hospital-based records | Live and stillborn infants weighing > 500g | Wheat flour | Folic acid | 2.2mg/kg |
| Noor [44] | 2017 | Tanzania | 2013 | Mandatory | Prospective cohort | Non-pregnant women of reproductive age (18-49 years) from two districts of Dar es Salaam | Wheat flour | Folic acid | 3 ± 2mg/kg |
| Pacheco [45] | 2009 | Brazil | 2004 | Mandatory | Retrospective live-birth or mortality information systems records | Live birth infants | Wheat Flour and maize flour | Folic acid | 1.5mg/kg |
| Ricks [46] | 2012 | Peru | 2006 | Mandatory | Retrospective hospital-based records | Live and stillborn infants | Wheat flour and bread | Folic acid | 1.21mg/kg to 2.19mg/kg |
| Sargiotto [47] | 2015 | Argentina | 2002 | Mandatory | Retrospective hospital-based records | Live births and stillbirths with a weight >500 grams, as reported by the RENAC and ECLAMC surveillance systems | Flour | Folic acid | 2.2 mg/kg |
| Sayed [48] | 2008 | South Africa | 2003 | Mandatory | Prospective hospital-based surveillance | Live and stillborn infants | Wheat (bread) and maize meal | Folic acid | Wheat: 1.5mg/kg  maize meal: 2.21mg.kg |
| Scorsatto [49] | 2011 |  |  |  |  |  |  |  |  |
| Wang [50] | 2016 | China | Not specified | Not specified | RCT | Women aged 18-35 years in Shanxi Province who were preparing for marriage or planning a pregnancy | Flour | Folic acid | 2 mg/kg |

**Supplemental Figure 1: Change in serum retinol (μg/dL) following LSFF with vitamin A.**

**
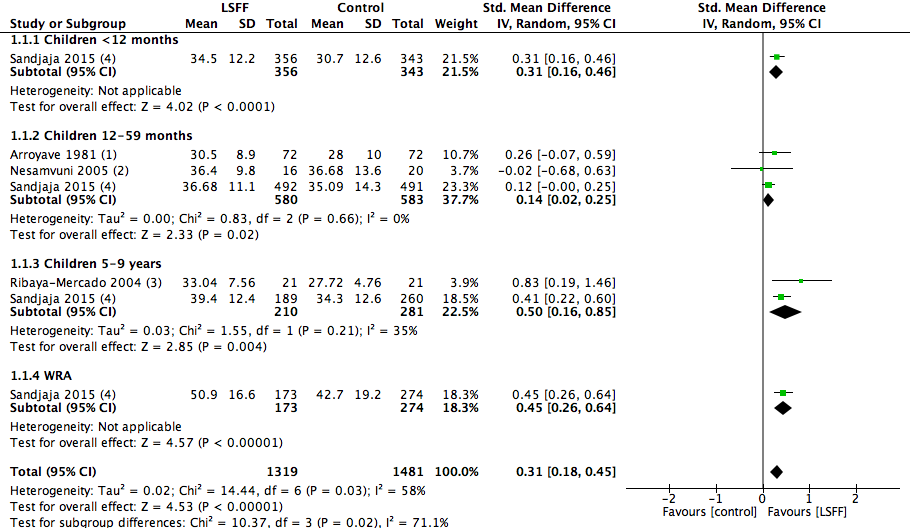
**

**Supplemental Figure 2: Urinary iodine (μg/dL) following salt iodization.**

**
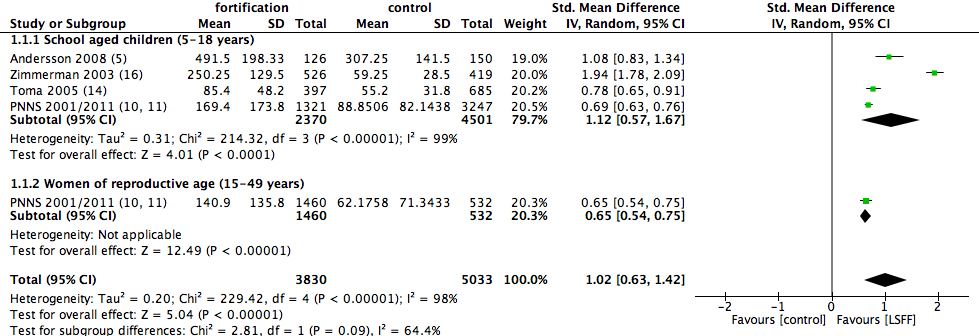
**

**Supplemental Figure 3: Prevalence of iodine deficiency following salt iodization.**

**
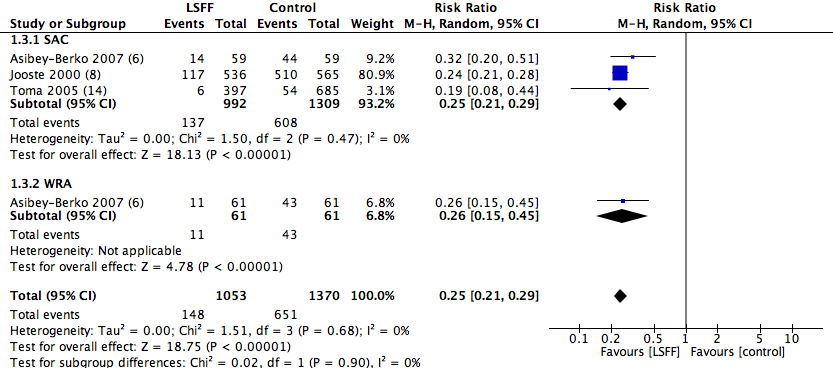
**

**Supplemental Figure 4: Change in haemoglobin concentration (g/dL) following LSFF with iron.**

**
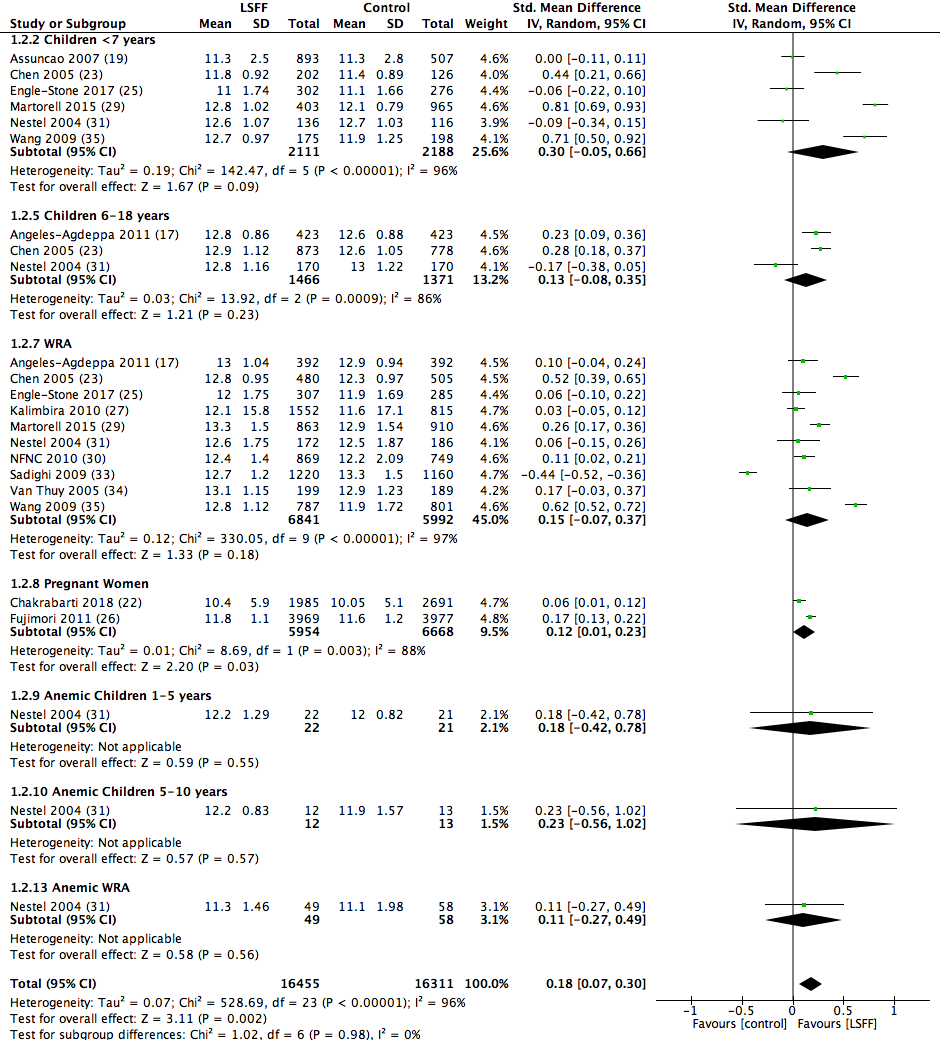
**

**Supplemental Figure 5: Prevalence of anemia following LSFF with iron.**

**
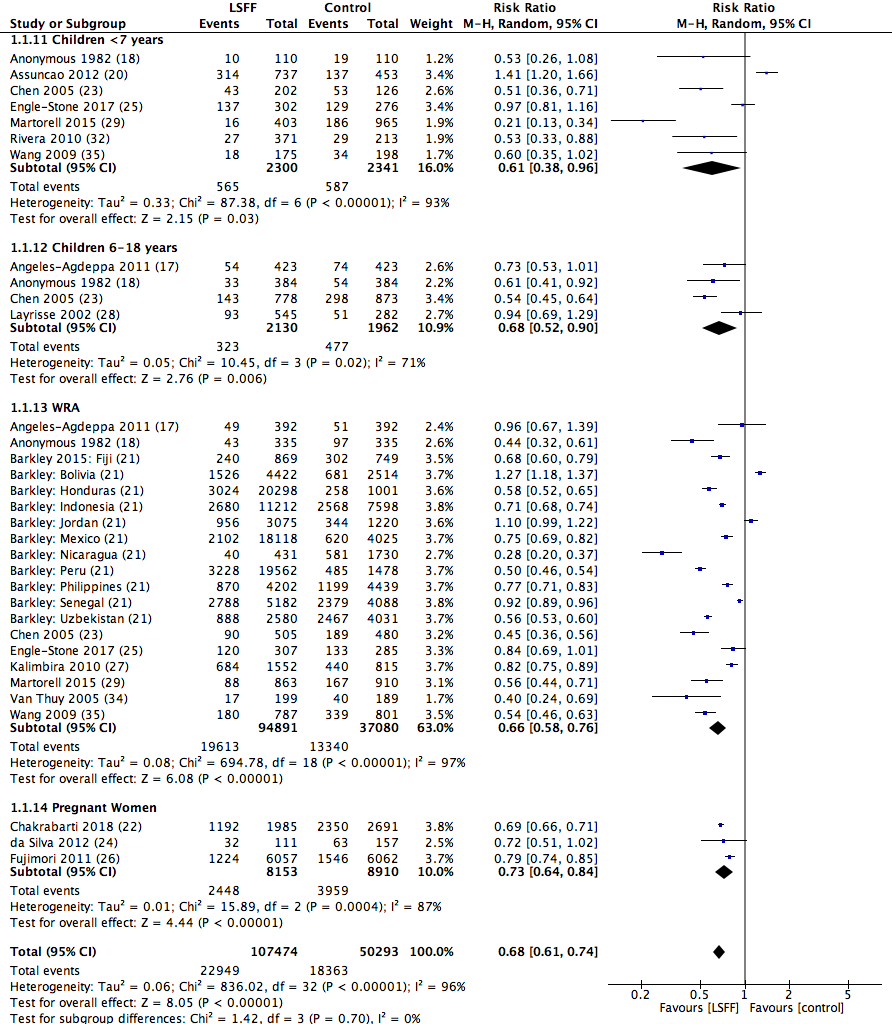
**

**Supplemental Figure 6: Change in serum ferritin (μg/L) following LSFF with iron.**

**
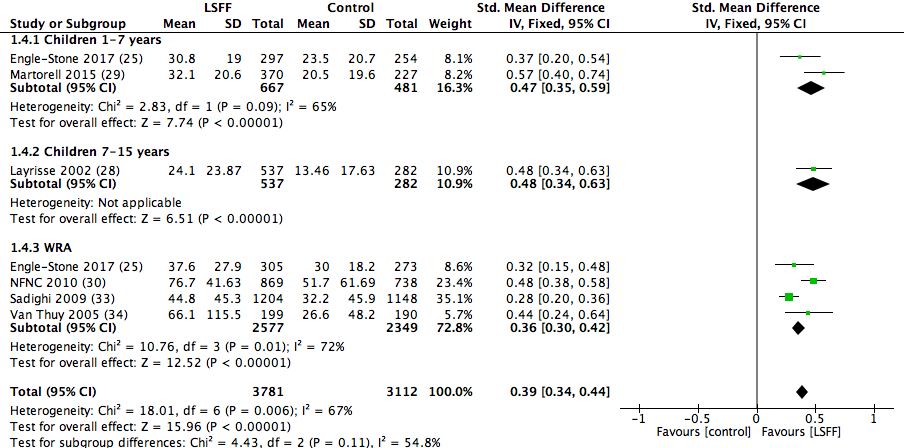
**

**Supplemental Figure 7: Prevalence of iron deficiency following LSFF with iron.**

**
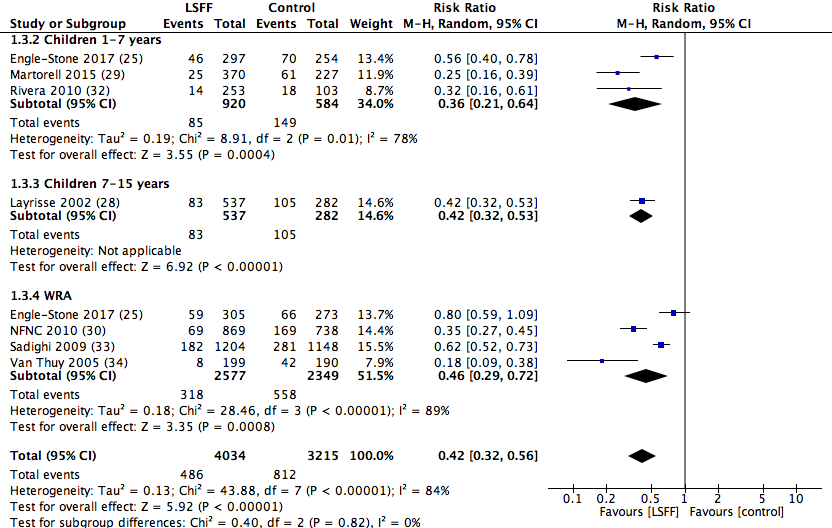
**

**Supplemental Figure 8: Prevalence of spina bifida following LSFF with folic acid.**

**
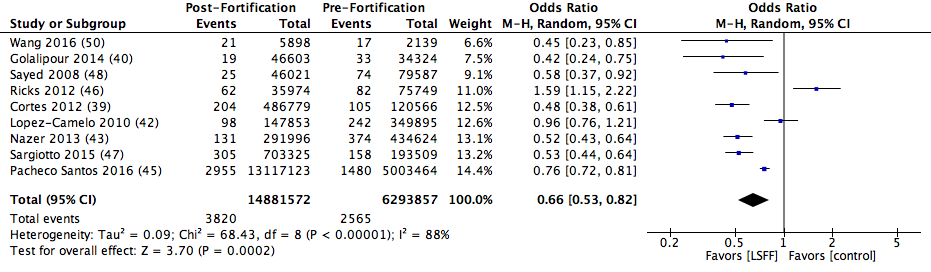
**

**Supplemental Figure 9: Prevalence of anencephaly following LSFF with folic acid.**

**
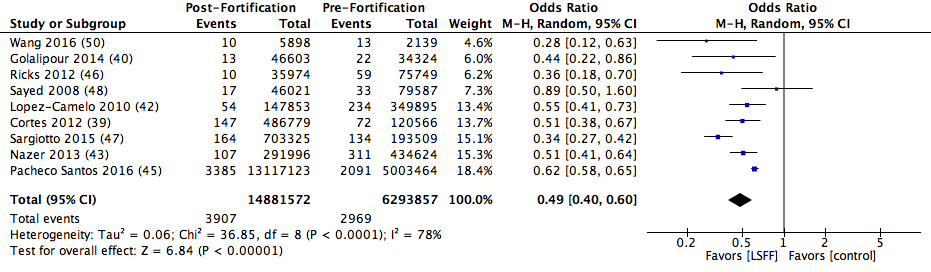
**

**Supplemental Figure 10: Prevalence of cephalocele following LSFF with folic acid.**

**
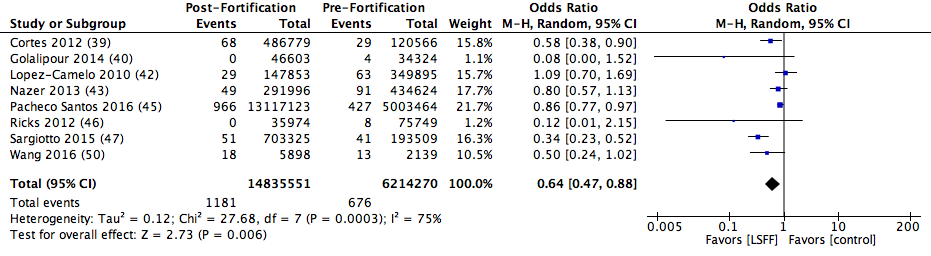
**

**Supplemental Figure 11: Prevalence of folate deficiency in WRA following LSFF with folic acid.**

**
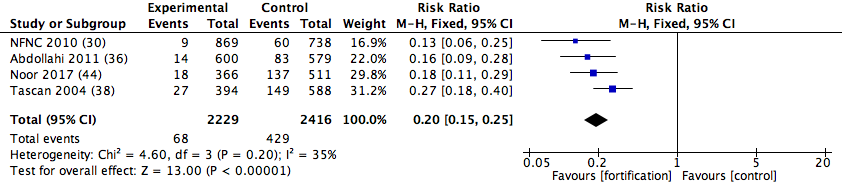
**

**Supplemental Figure 12: Change in serum/plasma folate (nmol/L) among WRA following LSFF with folic acid.**

**
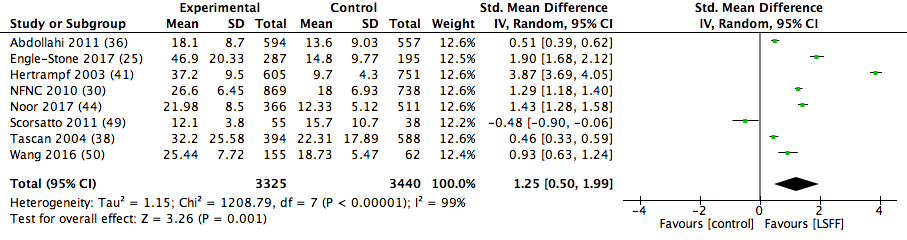
**

**References**

1. Arroyave G, Mejia LA, Aguilar JR. The effect of vitamin A fortification of sugar on the serum vitamin A levels of preschool Guatemalan children: a longitudinal evaluation. Am J Clin Nutr. 1981;34(1):41-9.

2. Nesamvuni AE, Vorster HH, Margetts BM, Kruger A. Fortification of maize meal improved the nutritional status of 1-3-year-old African children. Public Health Nutr. 2005;8(5):461-7.

3. Ribaya-Mercado JD, Solomons NW, Medrano Y, Bulux J, Dolnikowski GG, Russell RM, et al. Use of the deuterated-retinol-dilution technique to monitor the vitamin A status of Nicaraguan schoolchildren 1 y after initiation of the Nicaraguan national program of sugar fortification with vitamin A. Am J Clin Nutr. 2004;80(5):1291-8.

4. Sandjaja, Jus'at I, Jahari AB, Ifrad, Htet MK, Tilden RL, et al. Vitamin A-fortified cooking oil reduces vitamin A deficiency in infants, young children and women: results from a programme evaluation in Indonesia. Public Health Nutr. 2015;18(14):2511-22.

5. Andersson M, Thankachan P, Muthayya S, Goud RB, Kurpad AV, Hurrell RF, et al. Dual fortification of salt with iodine and iron: a randomized, double-blind, controlled trial of micronized ferric pyrophosphate and encapsulated ferrous fumarate in southern India. Am J Clin Nutr. 2008;88(5):1378-87.

6. Asibey-Berko E, Zlotkin SH, Yeung GS, Nti-Nimako W, Ahunu B, Kyei-Faried S, et al. Dual fortification of salt with iron and iodine in women and children in rural Ghana. East Afr Med J. 2007;84(10):473-80.

7. Erdogan MF, Demir O, Emral R, Kamel AN, Erdogan G. More than a decade of iodine prophylaxis is needed to eradicate goiter among school age children in a moderately iodine-deficient region. Thyroid. 2009;19(3):265-8.

8. Jooste PL, Weight MJ, Lombard CJ. Short-term effectiveness of mandatory iodization of table salt, at an elevated iodine concentration, on the iodine and goiter status of schoolchildren with endemic goiter. Am J Clin Nutr. 2000;71(1):75-80.

9. Mostafavi H. Effect of adequate salt iodization on the prevalence of goiter: a cross-sectional comparative study among school children aged 16-18 years during 1989 and 1995. Pak J Med Sci 2005;21(1):53-5.

10. Aga Khan University Pakistan. National Nutrition Survey 2001. Planning Commission and Planning and Development Division, Government of Pakistan 2001.

11. Aga Khan University Pakistan. National Nutrition Survey 2011. Planning Commission and Planning and Development Division, Government of Pakistan 2011.

12. Sooch SS, Ramalingaswami V. Preliminary Report of an Experiment in the Kangra Valley for the Prevention of Himalayan Endemic Goitre with Iodized Salt. Bull World Health Organ. 1965;32:299-315.

13. Sooch SS, Deo MG, Karmarkar MG, Kochupillai N, Ramachandran K, Ramalingaswami V. Prevention of endemic goitre with iodized salt. Bull World Health Organ. 1973;49(3):307-12.

14. Toma A. Universal salt iodization effects on endemic goiter in Arges County, Romania Acta Endocrinologica. 2005;1(2):167-80.

15. Wang P, Sun H, Shang L, Zhang Q, He Y, Chen Z, et al. Low Goiter Rate Associated with Small Average Thyroid Volume in Schoolchildren after the Elimination of Iodine Deficiency Disorders. PLoS One. 2015;10(10):e0141552.

16. Zimmermann MB, Hess SY, Adou P, Toresanni T, Wegmuller R, Hurrell RF. Thyroid size and goiter prevalence after introduction of iodized salt: a 5-y prospective study in schoolchildren in Cote d'Ivoire. Am J Clin Nutr. 2003;77(3):663-7.

17. Angeles-Agdeppa I, Saises M, Capanzana M, Juneja LR, Sakaguchi N. Pilot-scale commercialization of iron-fortified rice: effects on anemia status. Food Nutr Bull. 2011;32(1):3-12.

18. Use of common salt fortified with iron in the control and prevention of anemia--a collaborative study. Report of the Working Group on Fortification of Salt with Iron. Am J Clin Nutr. 1982;35(6):1442-51.

19. Assuncao MC, Santos IS, Barros AJ, Gigante DP, Victora CG. [Effect of iron fortification of flour on anemia in preschool children in Pelotas, Brazil]. Rev Saude Publica. 2007;41(4):539-48.

20. Assuncao MC, Santos IS, Barros AJ, Gigante DP, Victora CG. Flour fortification with iron has no impact on anaemia in urban Brazilian children. Public Health Nutr. 2012;15(10):1796-801.

21. Barkley JS, Wheeler KS, Pachon H. Anaemia prevalence may be reduced among countries that fortify flour. Br J Nutr. 2015;114(2):265-73.

22. Chakrabarti S, Kishore A, Raghunathan K, Scott SP. Impact of subsidized fortified wheat on anaemia in pregnant Indian women. Maternal & child nutrition. 2018:e12669.

23. Chen J, Zhao X, Zhang X, Yin S, Piao J, Huo J, et al. Studies on the effectiveness of NaFeEDTA-fortified soy sauce in controlling iron deficiency: a population-based intervention trial. Food Nutr Bull. 2005;26(2):177-86; discussion 87-9.

24. da Silva CL, Saunders C, Szarfarc SC, Fujimori E, da Veiga GV. Anaemia in pregnant women before and after the mandatory fortification of wheat and corn flours with iron. Public Health Nutr. 2012;15(10):1802-9.

25. Engle-Stone R, Nankap M, Ndjebayi AO, Allen LH, Shahab-Ferdows S, Hampel D, et al. Iron, Zinc, Folate, and Vitamin B-12 Status Increased among Women and Children in Yaounde and Douala, Cameroon, 1 Year after Introducing Fortified Wheat Flour. The Journal of nutrition. 2017;147(7):1426-36.

26. Fujimori E, Sato AP, Szarfarc SC, Veiga GV, Oliveira VA, Colli C, et al. Anemia in Brazilian pregnant women before and after flour fortification with iron. Rev Saude Publica. 2011;45(6):1027-35.

27. Kalimbira AA, MacDonald C, Simpson JR. The impact of an integrated community-based micronutrient and health programme on anaemia in non-pregnant Malawian women. Public Health Nutr. 2010;13(9):1445-52.

28. Layrisse M, Garcia-Casal MN, Mendez-Castellano H, Jimenez M, Henry O, Chavez JE, et al. Impact of fortification of flours with iron to reduce the prevalence of anemia and iron deficiency among schoolchildren in Caracas, Venezuela: a follow-up. Food Nutr Bull. 2002;23(4):384-9.

29. Martorell R, Ascencio M, Tacsan L, Alfaro T, Young MF, Addo OY, et al. Effectiveness evaluation of the food fortification program of Costa Rica: impact on anemia prevalence and hemoglobin concentrations in women and children. Am J Clin Nutr. 2015;101(1):210-7.

30. National Food and Nutrition Centre. Impact of Iron Fortified Flour in Child Bearing Age (CBA) Women in Fiji: 2010 Report. Suva, Fiji; 2012.

31. Nestel P, Nalubola R, Sivakaneshan R, Wickramasinghe AR, Atukorala S, Wickramanayake T. The use of iron-fortified wheat flour to reduce anemia among the estate population in Sri Lanka. Int J Vitam Nutr Res. 2004;74(1):35-51.

32. Rivera JA, Shamah T, Villalpando S, Monterrubio E. Effectiveness of a large-scale iron-fortified milk distribution program on anemia and iron deficiency in low-income young children in Mexico. Am J Clin Nutr. 2010;91(2):431-9.

33. Sadighi J, Mohammad K, Sheikholeslam R, Amirkhani MA, Torabi P, Salehi F, et al. Anaemia control: lessons from the flour fortification programme. Public Health. 2009;123(12):794-9.

34. Van Thuy P, Berger J, Nakanishi Y, Khan NC, Lynch S, Dixon P. The use of NaFeEDTA-fortified fish sauce is an effective tool for controlling iron deficiency in women of childbearing age in rural Vietnam. J Nutr. 2005;135(11):2596-601.

35. Wang B, Zhan S, Sun J, Lee L. Social mobilization and social marketing to promote NaFeEDTA-fortified soya sauce in an iron-deficient population through a public-private partnership. Public Health Nutr. 2009;12(10):1751-9.

36. Abdollahi Z, Elmadfa I, Djazayery A, Golalipour MJ, Sadighi J, Salehi F, et al. Efficacy of flour fortification with folic acid in women of childbearing age in Iran. Ann Nutr Metab. 2011;58(3):188-96.

37. Amarin ZO, Obeidat AZ. Effect of folic acid fortification on the incidence of neural tube defects. Paediatr Perinat Epidemiol. 2010;24(4):349-51.

38. Chen LT, Rivera MA. The Costa Rican experience: reduction of neural tube defects following food fortification programs. Nutr Rev. 2004;62(6 Pt 2):S40-3.

39. Cortes F, Mellado C, Pardo RA, Villarroel LA, Hertrampf E. Wheat flour fortification with folic acid: changes in neural tube defects rates in Chile. Am J Med Genet A. 2012;158A(8):1885-90.

40. Golalipour MJ, Arabi M, Mohammad A. Impact of flour fortification with folic acid on the prevalence of neural tube defects in Northern Iran. Journal of Pediatric Neurology 2014;12:69-73.

41. Hertrampf E, Cortes F, Erickson JD, Cayazzo M, Freire W, Bailey LB, et al. Consumption of folic acid-fortified bread improves folate status in women of reproductive age in Chile. J Nutr. 2003;133(10):3166-9.

42. Lopez-Camelo JS, Castilla EE, Orioli IM, Inagemp, Eclamc. Folic acid flour fortification: impact on the frequencies of 52 congenital anomaly types in three South American countries. Am J Med Genet A. 2010;152A(10):2444-58.

43. Nazer NH, Cifuentes LO. Results of the prevention of neural tube defects in Chile through fortification of flour with folic acid. Period 2001-2010. Chilean Medical Journal 2013;141(6).

44. Noor RA, Abioye AI, Ulenga N, Msham S, Kaishozi G, Gunaratna NS, et al. Large -scale wheat flour folic acid fortification program increases plasma folate levels among women of reproductive age in urban Tanzania. PloS one. 2017;12(8):e0182099.

45. Pacheco SS, Braga C, Souza AI, Figueiroa JN. Effects of folic acid fortification on the prevalence of neural tube defects. Rev Saude Publica. 2009;43(4):565-71.

46. Ricks DJ, Rees CA, Osborn KA, Crookston BT, Leaver K, Merrill SB, et al. Peru's national folic acid fortification program and its effect on neural tube defects in Lima. Rev Panam Salud Publica. 2012;32(6):391-8.

47. Sargiotto C, Bidondo MP, Liascovich R, Barbero P, Groisman B. Descriptive study on neural tube defects in Argentina. Birth Defects Res A Clin Mol Teratol. 2015;103(6):509-16.

48. Sayed AR, Bourne D, Pattinson R, Nixon J, Henderson B. Decline in the prevalence of neural tube defects following folic acid fortification and its cost-benefit in South Africa. Birth Defects Res A Clin Mol Teratol. 2008;82(4):211-6.

49. Scorsatto M, Uehara SK, Luiz RR, de Oliveira GM, Rosa G. Fortification of flours with folic acid reduces homocysteine levels in Brazilian women. Nutr Res. 2011;31(12):889-95.

50. Wang H, De Steur H, Chen G, Zhang X, Pei L, Gellynck X, et al. Effectiveness of Folic Acid Fortified Flour for Prevention of Neural Tube Defects in a High Risk Region. Nutrients. 2016;8(3):152.
